# Supplementary material for: Description of two new species and a new population of Mesobiotus cf. coronatus from Cotacachi-Cayapas National Park, Ecuador
Source: PLoS One. 2025 Jun 4;20(6):e0324518. doi: 10.1371/journal.pone.0324518 (PMC12136313; doi:10.1371/journal.pone.0324518)
Supplement: Attachment — Submitted filename: PONE-D-25-06719_reviewer_REv.pdf [file pone.0324518.s008.pdf]

PLOS ONE

An integrative description of three new eutardigrade species from Cotacachi-Cayapas National Park, Ecuador

--Manuscript Draft--

**Manuscript Number:**	PONE-D-25-06719
**Article Type:**	Research Article
**Full Title:**	An integrative description of three new eutardigrade species from Cotacachi-Cayapas National Park, Ecuador
**Short Title:**	Three new eutardigrades from Ecuador
**Corresponding Author:**	Anastasiia Polishchuk, MSc<br>Adam Mickiewicz University: Uniwersytet im Adama Mickiewicza w Poznaniu<br>Poznań, POLAND
**Keywords:**	Tardigrada, Macrobiotidae, Ramazzottiidae, water bears, taxonomy, new species
**Abstract:**	In this study, we present descriptions of three new eutardigrade species discovered in the Cotacachi-Cayapas National Park in Ecuador. The presented analysis is based on morphological and ~~morphometrical~~ data using light and scanning electron microscopy; description of Macrobiotus sharopovi sp. nov. and Mesobiotus shevchenkoi sp. nov. were also supported with genetic data (nuclear barcode sequences, i.e. 18S rRNA, 28S rRNA and ITS-2, and one mitochondrial COI sequences). Based on the egg morphology, Mac. sharopovi sp. nov. belongs to Mac. paulinae morpho-group and is most similar to Mac. papei, Mac. paulinae, Mac. polypiformis and Mac. shonaicus but differs mainly by some characters of eggs (number of egg processes on circumference, details of eggshell morphology and features of filaments and their number) and adults (size of cuticular pores). Mesobiotus shevchenkoi sp. nov. belongs to Meb. harmsworthi morpho-group and in egg morphology is most similar to Meb. coronatus, Meb. insuetus, Meb. occultatus, Meb. patiens, Meb. philippinicus, Meb. pseudocoronatus, Meb. simulans but differs mainly by egg and egg processes size as well as their number on circumference. Based on such features as the presence of gibbosities and well visible dorsal sculpture, third new species i.e. Ramazzottius syraxi sp. nov. is the most similar to Ram. saltensis and Ram. szeptycki but differs by gibbosities configuration and characteristic of dorsal sculpture.
**Order of Authors:**	Anastasiia Polishchuk, MSc
	Pushpalata Kayastha, PhD
	Dominika Młodzianowska, BSc
	Martyna Michalska, BSc
	Magdalena Gawlak, PhD
	Jędrzej Warguła, BSc
	Łukasz Kaczmarek, PhD
**Additional Information:**	
**Question**	**Response**
**Financial Disclosure**<br><br>Enter a financial disclosure statement that describes the sources of funding for the work included in this submission. Review the submission guidelines for detailed requirements. View published research articles from *PLOS ONE* for specific examples.	Yes

Powered by Editorial Manager® and ProduXion Manager® from Aries Systems Corporation

This statement is required for submission and **will appear in the published article** if the submission is accepted. Please make sure it is accurate.<br><br>**Funded studies**<br>Enter a statement with the following details:<br>• Initials of the authors who received each award<br>• Grant numbers awarded to each author<br>• The full name of each funder<br>• URL of each funder website<br>• Did the sponsors or funders play any role in the study design, data collection and analysis, decision to publish, or preparation of the manuscript?<br><br>Did you receive funding for this work?	
Please add funding details.<br>as follow-up to "**Financial Disclosure**<br><br>Enter a financial disclosure statement that describes the sources of funding for the work included in this submission. Review the submission guidelines for detailed requirements. View published research articles from *PLOS ONE* for specific examples.<br><br>This statement is required for submission and **will appear in the published article** if the submission is accepted. Please make sure it is accurate.<br><br>**Funded studies**<br>Enter a statement with the following details:<br>• Initials of the authors who received each award<br>• Grant numbers awarded to each author<br>• The full name of each funder<br>• URL of each funder website<br>• Did the sponsors or funders play any role in the study design, data collection and analysis, decision to publish, or preparation of the manuscript?	The work of Anastasiia Polishchuk was supported by grant no. 2022/01/4/NZ4/00009 from the National Science Centre (Poland)

Powered by Editorial Manager® and ProduXion Manager® from Aries Systems Corporation

Did you receive funding for this work?"	
Please select the country of your main research funder (please select carefully as in some cases this is used in fee calculation).<br>as follow-up to "**Financial Disclosure**<br><br>Enter a financial disclosure statement that describes the sources of funding for the work included in this submission. Review the submission guidelines for detailed requirements. View published research articles from *PLOS ONE* for specific examples.<br><br>This statement is required for submission and **will appear in the published article** if the submission is accepted. Please make sure it is accurate.<br><br>**Funded studies**<br>Enter a statement with the following details:<br>• Initials of the authors who received each award<br>• Grant numbers awarded to each author<br>• The full name of each funder<br>• URL of each funder website<br>• Did the sponsors or funders play any role in the study design, data collection and analysis, decision to publish, or preparation of the manuscript?<br><br>Did you receive funding for this work?"	POLAND - PL
**Competing Interests**<br><br>Use the instructions below to enter a competing interest statement for this submission. On behalf of all authors, disclose any competing interests that could be perceived to bias this work—acknowledging all financial support and any other relevant financial or non-financial competing interests.<br><br>This statement is required for submission	The authors have declared that no competing interests exist.

and **will appear in the published article** if the submission is accepted. Please make sure it is accurate and that any funding sources listed in your Funding Information later in the submission form are also declared in your Financial Disclosure statement.<br><br>View published research articles from *PLOS ONE* for specific examples.<br><br>**NO authors have competing interests**<br><br>Enter: *The authors have declared that no competing interests exist.*<br><br>**Authors with competing interests**<br><br>Enter competing interest details beginning with this statement:<br><br>*I have read the journal's policy and the authors of this manuscript have the following competing interests: [insert competing interests here]*<br><br>* typeset	
**Ethics Statement**<br><br>Enter an ethics statement for this submission. This statement is required if the study involved:<br><br>• Human participants<br>• Human specimens or tissue<br>• Vertebrate animals or cephalopods<br>• Vertebrate embryos or tissues<br>• Field research<br><br>Write "N/A" if the submission does not require an ethics statement.<br><br>General guidance is provided below. Consult the submission guidelines for detailed instructions. **Make sure that all information entered here is included in the Methods section of the manuscript.**	Samples were collected in Cotacachi-Cayapas National Park (Imbabura Province, Ecuador) in 2014 with the permit №001-15IC-FLO-FAU-DNB/MA

**Format for specific study types**<br><br>**Human Subject Research (involving human participants and/or tissue)**<br>• Give the name of the institutional review board or ethics committee that approved the study<br>• Include the approval number and/or a statement indicating approval of this research<br>• Indicate the form of consent obtained (written/oral) or the reason that consent was not obtained (e.g. the data were analyzed anonymously)<br><br>**Animal Research (involving vertebrate animals, embryos or tissues)**<br>• Provide the name of the Institutional Animal Care and Use Committee (IACUC) or other relevant ethics board that reviewed the study protocol, and indicate whether they approved this research or granted a formal waiver of ethical approval<br>• Include an approval number if one was obtained<br>• If the study involved *non-human primates*, add *additional details* about animal welfare and steps taken to ameliorate suffering<br>• If anesthesia, euthanasia, or any kind of animal sacrifice is part of the study, include briefly which substances and/or methods were applied<br><br>**Field Research**<br><br>Include the following details if this study involves the collection of plant, animal, or other materials from a natural setting:<br>• Field permit number<br>• Name of the institution or relevant body that granted permission	
**Data Availability**<br><br>Authors are required to make all data underlying the findings described fully available, without restriction, and from the time of publication. PLOS allows rare exceptions to address legal and ethical concerns. See the PLOS Data Policy and FAQ for detailed information.	Yes - all data are fully available without restriction

A Data Availability Statement describing where the data can be found is required at submission. Your answers to this question constitute the Data Availability Statement and **will be published in the article**, if accepted.<br><br>**Important:** Stating 'data available on request from the author' is not sufficient. If your data are only available upon request, select 'No' for the first question and explain your exceptional situation in the text box.<br><br>Do the authors confirm that all data underlying the findings described in their manuscript are fully available without restriction?	
**Describe where the data may be found in full sentences. If you are copying our sample text, replace any instances of XXX with the appropriate details.**<br><br>• If the data are **held or will be held in a public repository**, include URLs, accession numbers or DOIs. If this information will only be available after acceptance, indicate this by ticking the box below. For example: *All XXX files are available from the XXX database (accession number(s) XXX, XXX.*).<br>• If the data are all contained **within the manuscript and/or Supporting Information files**, enter the following: *All relevant data are within the manuscript and its Supporting Information files.*<br>• If neither of these applies but you are able to provide **details of access elsewhere**, with or without limitations, please do so. For example:<br><br>*Data cannot be shared publicly because of [XXX]. Data are available from the XXX Institutional Data Access / Ethics Committee (contact via XXX) for researchers who meet the criteria for access to confidential data.*<br><br>*The data underlying the results presented in the study are available from (include the name of the third party*	All relevant data are within the manuscript and its Supporting Information files.

and contact information or URL).<br>• This text is appropriate if the data are owned by a third party and authors do not have permission to share the data.<br><br>* typeset	
Additional data availability information:	

Manuscript

Click here to access/download;Manuscript;Three_new_eutardigrades_from_

~~An~~ integrative description of three new eutardigrade species from Cotacachi-Cayapas National Park, Ecuador

Anastasiia Polishchuk[1*], Pushpalata Kayastha[2], Dominika Młodzianowska[3], Martyna Michalska[3], Magdalena Gawlak[4], Jędrzej Warguła[1], Łukasz Kaczmarek[1]

[1]Department of Animal Taxonomy and Ecology, Adam Mickiewicz University in Poznań, Poznań, Poland

[2]Biology Department, The University of North Carolina at Chapel Hill, Chapel Hill, NC, USA

[3]Department of Genetics and Biosystematics, Faculty of Biology, University of Gdańsk, Gdańsk, Poland

[4]Institute of Plant Protection – National Research Institute, Poznań, Poland

* Corresponding author

E-mail: anastasiia.m.polishchuk@gmail.com (AP)

Short title: Three new eutardigrades from Ecuador

Abstract

In this study, we present descriptions of three new eutardigrade species discovered in the Cotacachi-Cayapas National Park in Ecuador. The presented analysis is based on morphological and ~~morphometrical~~ data using light and scanning electron microscopy; description of *Macrobiotus sharopovi* sp. nov. and *Mesobiotus shevchenkoi* sp. nov. were also supported with genetic data (nuclear barcode sequences, i.e. 18S rRNA, 28S rRNA and ITS-2, and one mitochondrial COI sequences). Based on the egg morphology, *Mac. sharopovi* sp. nov. belongs to *Mac. paulinae* morpho-group and is most similar to *Mac. papei, Mac. paulinae, Mac. polypiformis* and *Mac. shonaicus* but differs mainly by some characters of eggs (number of egg processes on circumference, details of eggshell morphology and features of filaments and their number) and adults (size of cuticular pores). *Mesobiotus shevchenkoi* sp. nov. belongs to *Meb. harmsworthi* morpho-group and in egg morphology is most similar to *Meb. coronatus, Meb. insuetus, Meb. occultatus, Meb. patiens, Meb. philippinicus, Meb. pseudocoronatus, Meb. simulans* but differs mainly by egg and egg processes size as well as their number on circumference. Based on such features as the presence of gibbosities and well visible dorsal sculpture, third new species i.e. *Ramazzottius syraxi* sp. nov. is the most similar to *Ram. saltensis* and *Ram. szeptycki* but differs by gibbosities configuration and characteristic of dorsal sculpture.

Introduction

The Cotacachi-Cayapas National Park, established in 2019, originated from the Cotacachi-Cayapas Ecological Reserve, which was erected in 1968. It is located in the northwestern part of Ecuador and spans the provinces of Esmeraldas (San Lorenzo, Eloy Alfaro

and Río Verde cantons) and Imbabura (Cotacachi, Urcuquí and Ibarra cantons), with a total area of 260,961 ha [1].

The Cotacachi-Cayapas National Park is one of the largest protected areas in the country with exceptional biodiversity and varied ecosystems. It extends from the high-altitude Andean region covered by tundra-like vegetation (páramo) with the notable geographical feature Cotacachi Volcano (4989 m asl) on the west to the lowland tropical rainforests with swamps and lagoons on the east. That eastern part belongs to the Chocó biogeographic region, which in turn is a part of the Tumbes-Chocó-Magdalena biodiversity hotspot, one of the world's most species-rich areas [2].

The variety of natural environments and the high-altitude range in the park contribute to the existence of a high diversity of species of flora and fauna e.g. 2225 species of plants, 139 species of mammals, 845 bird species, 111 reptiles, 124 amphibians and 39 fish species with showcasing a high level of endemism [1]. At the same time, Cotacachi-Cayapas National Park is a focus for conservationists due to the threats of deforestation, mining and climate change, especially the rainforests of the Chocó region [2,3].

The phylum Tardigrada includes over 1400 described species of microscopic invertebrates [4] noted for their exceptional resilience across diverse environmental conditions [5,6]. These organisms inhabit terrestrial, freshwater and marine ecosystems worldwide, and can be found in mosses, lichens, soil, leaf litter, as well as in/on marine and freshwater sediments and plants [7].

The family Macrobiotidae Thulin 1928 [8], consisting of limno-terrestrial tardigrades, encompasses 14 recognized genera. Notably, a significant portion of species diversity within this family is concentrated in four genera: *Macrobiotus* Schultze 1834 [9], *Mesobiotus* Vecchi, Cesari, Bertolani, Jönsson, Rebecchi & Guidetti 2016 [10], *Minibiotus* Schuster 1980 [11] and *Paramacrobiotus* Guidetti, Schill, Bertolani, Dandekar & Wolf 2009 [12]. These genera were

initially identified as informal species groups under *Macrobiotus* and were subsequently elevated to genus status through successive taxonomic reassessments [10-12]. This classification reflects the evolving understanding of their phylogenetic relationships and ecological significance.

The genus *Macrobiotus*, established by Schultze [9], has one of the most complex revision histories within the phylum Tardigrada, reflecting its extensive phenotypic diversity, especially in egg ornamentation morphology [13-16]. This complexity has prompted numerous studies aimed at refining the definition of the genus and clarifying the classification of smaller groups of species within it [14-21]. Despite these efforts, reaching a consensus has been challenging due to the vast morphological diversity of the genus, which complicates the establishment of clear, strict definitions that also consider phylogenetic relationships. Current studies support the internal division of *Macrobiotus* into three distinct, deeply divergent phylogenetic lineages – clades A, B, and C [15,19-21]. Within these clades, several smaller groups have achieved recognition as monophyletic and have been defined as species complexes, such as the *Mac. pallarii*, *Mac. pseudohufelandi*, *Mac. ariekammensis* and *Mac. polonicus* complexes, with the latter, recently redefined as the *Mac. polonicus-persimilis* complex [21]. Other taxa within the genus have been organized into informal, non-monophyletic species morpho-groups, including the *Mac. hufelandi* and newly redefined *Mac. nelsonae* morpho-groups [16]. Despite these categorizations, the subdivision of *Macrobiotus* remains complex, prompting Kaczmarek et al. [16] to propose an artificial division of all taxa into 12 morphological groups based solely on eggshell morphology, potentially simplifying taxonomic research and facilitating better navigation among taxa, although the long-term acceptance and utility of this methodology remains to be seen.

The genus *Mesobiotus* encompasses 79 nominal species, including four designated as *nomina inquirenda* [4,22]. Established by Vecchi et al. [10], *Mesobiotus* is supported by both

morphological and genetic evidence. Research indicates that the genus is monophyletic; however, it does not support the division into the two traditionally recognized species groups – *harmsworthi* and *furciger* – which are distinguished primarily by the morphology of egg processes (specifically, the *furciger* group is characterized by dichotomous branching at the tips of the egg processes, this feature is absent in the *harmsworthi* group). Due to the lack of reciprocal monophyly, Short et al. [23] suggested that these informal groups should be discontinued. In turn, Stec [24] advocates for retaining these morpho-groups for their utility in taxonomic identification and communication. Furthermore, Stec [24] redefined the *harmsworthi* and *furciger* morpho-groups with greater precision and introduced a new morpho-group – *montanus,* characterized by dome-shaped egg processes, to enhance classification accuracy and clarity in taxonomic discourse.

In the family Ramazzottiidae Sands, McInnes, Marley, Goodall-Copestake, Convey & Linse 2008 [25], genus *Ramazzottius* Binda & Pilato 1986 [26] encompasses 30 species [4], including the type species *Ramazzottius oberhaeuseri* [27], and characterized by the presence of extremely different in size and shape internal and external claws, apophyses for the insertion of the stylet muscles in the shape of “blunt hooks” and general presence of a paired elliptical organs on the head [26,28]. Species of this genus are often found in xeric mosses and lichens and are one of the most common and distributed globally eutardigrades [29-33]. Despite this, the genus *Ramazzottius* includes various species complexes with many taxonomic problems and not fully resolved phylogenetic relationships [34,35]. For example, polyphyletic *oberhaeuseri* morpho-group (eggs with hemispherical processes and cryptic and pseudocryptic speciation) [36,37] and the monophyletic complexes *baumanni* and *szeptyckii* (peculiar, ornamented cuticle and unknown egg morphology) [35]. This situation has occurred due to a lack of genetic data and mostly outdated descriptions of the species [35].

The tardigrade fauna of Ecuador is one of the poorest studied not only in South America, but also in the world. So far, only 27 species were reported from mainland Ecuador [31,38-41] and many of them are now considered as species complexes or doubtful taxa (for more details see e.g. Kaczmarek et al. [31]). This suggests that tardigrades in this region are very poorly known, and many taxa await discovery.

In this paper, we are describing three new eutardigrade species from Ecuador: *Macrobiotus sharopovi* sp. now. (*Mac. hufelandi* morpho-group), *Mesobiotus shevchenkoi* sp. nov. (*Meb. harmsworthi* morpho-group) and *Ramazzottius syraxi* sp. nov.

Material and methods

Samples and sample processing

Forty-four samples of mosses, lichens, cryptograms and algae were collected from soil, trees, rocks, shrubs, dead wood and concrete wall on December 16–17, 2014 by Milena Roszkowska and Łukasz Kaczmarek in the south of the Cotacachi-Cayapas National Park (Imbabura Province, Ecuador) (permit №001-15IC-FLO-FAU-DNB/MA).

Samples were packed in paper envelopes, dried at a temperature of *ca.* 20°C and delivered to the laboratory at the Faculty of Biology, Adam Mickiewicz University in Poznań, Poland. Tardigrades and their eggs were extracted from the samples and studied following the protocol in Stec et al. [42].

Microscopy and imaging

In total 158 specimens and 49 eggs (90 specimens and 19 eggs of *Mac. sharopovi* sp. nov., 55 specimens and 30 eggs of *Meb. shevchenkoi* sp. nov. and 13 specimens of *Ram. syraxi* sp. nov. (seven specimens from the type locality and six specimens from the additional

locality)) were mounted on microscope slides in Hoyer's medium and secured with a cover slips. Then slides were dried in the heater for two days at *ca.* 60°C and sealed with transparent nail polish. Prepared slides were examined with an Olympus BX41 phase-contrast light microscope (PCM) associated with an ART-CAM–300Mi digital camera (Olympus Corporation, Shin-juku–ku, Japan) for imaging.

An additional 38 specimens and 10 eggs of *Mac. sharopovi* sp. nov. and 25 specimens and 10 eggs of *Meb. shevchenkoi* sp. nov. were prepared for Scanning Electron Microscope (SEM) analysis according to the protocol in Roszkowska et al. [40] and examined under high vacuum in Hitachi S3000N SEM (Hitachi, Japan).

All figures were assembled in Corel Photo-Paint 2021. For deep structures that could not be fully focused on a single photograph, a series of 2–10 images were taken every *ca.* 0.5 micrometers [μm] and then assembled into a single deep-focus image manually in the above-mentioned program.

Morphometrics and morphological nomenclature

All measurements are given in micrometers [μm]. Structures were measured only if their orientation was suitable. Body length was measured from the anterior extremity to the end of the body, excluding the hind legs.

For *Macrobiotus* and *Ramazzottius*, the classification of the buccal apparatus and claws follows the system developed by Pilato and Binda [28] and amended for *Mesobiotus* by Vecchi et al. [10]. Terminology for the oral cavity armature (OCA) and eggshell morphology is in accordance with Michalczyk and Kaczmarek [43], Stec et al. [36] and Kaczmarek and Michalczyk [14]. Additionally, the sequence of macroplacoid lengths is determined based on Kaczmarek et al. [44], while the morphological states of the cuticular bars on legs are categorized following Kiosya et al. [45]. All other measurements and the nomenclature

employed adhere to the guidelines provided by Beasley et al. [46], Stec et al. [36] and Kaczmarek and Michalczyk [14].

The *pt* index, which is defined as the ratio of the length of a given structure to the length of the buccal tube expressed as a percentage according to Pilato [47].

The measurements were performed and recorded according to the “Parachela” using the “Parachela” ver. 1.8 template available from the Tardigrada Register [48] with modification by Massa et al. [17] for Thorpe’s Normalization. Raw morphometric data for the specimens of all new described species are given in Supporting Information (S1–S4 Tables).

Genus’s abbreviations follow Perry et al. [49].

DNA extraction and genotyping

Twenty specimens of *Mac. sharopovi* sp. nov. and seven specimens of *Meb. shevchenkoi* sp. nov. were prepared for genetic analyses, out of which barcodes from three specimens of *Mac. sharopovi* sp. nov. and one specimen of *Meb. shevchenkoi* sp. nov. were obtained in good quality and used for analysis.

Light microscopy (LM) was used for *in vivo* specimen identification prior to genomic DNA extraction. In order to obtain voucher specimens, DNA extractions from individuals were performed using a Chelex® 100 resin (Bio-Rad) extraction method supplied by Casquet et al. [50], with modifications outlined in Stec et al. [42]. Exoskeletons were mounted on microscope slides with Hoyer’s medium. Four DNA segments were attempted to be sequenced: one mitochondrial (COI) and three nuclear (18S rRNA, 28S rRNA, and ITS2) using following primers: HCO2198 (5′–TAAACTTCAGGGTGACCAAAAAATCA–3′) and LCO1490 (5′–GGTCAACAAATCATAAAGATATTGG–3′) [51] for the *cox1* gene fragment; SSU01_F (5′–AACCTGGTTGATCCTGCCAGT–3′) and SSU82_R (5′–TGATCCTT CTGCAGGTTCACCTAC–3′) [25] for the 18S rRNA gene fragment; 28SF0001 (5′–

ACCCvCynAATTTAAGCATAT–3′) and 28SR 0990 (5′–CCTTGGTCCGTGTTTCAAGAC–3′) [52] for the 28S rRNA gene fragment; ITS-2 Eutar_Ff (5′– CGTAACGTGAATTGCAGGAC–3′) and ITS-2 Eutar_Rr (5′–TCCTCCGCTTATTGATATGC–3′) [53] for the ITS-2 gene fragment. All the molecular markers underwent amplification using protocol by Kaczmarek et al. [54]. PCR products were purified with thermosensitive Exonuclease I and FastAP Alkaline Phosphatase (Thermo Scientific) and sequenced with BigDye Terminator v3.1 on an ABI Prism 3130XL Analyzer (Applied Biosystems) according to manufacturer's instructions. Sequence chromatograms were checked for accuracy using FinchTV 1.3.1 (Geospiza Inc.)

For comparative molecular analysis, sequence homology and identity were confirmed using the Basic Local Alignment Search Tool (BLAST) [55]. Sequence alignments were performed using the AUTO method for COI and ITS-2 markers, and the Q-INS-I method for ribosomal markers (18S rRNA and 28S rRNA) in MAFFT version 7 [56,57]. The alignments were then manually verified for non-conserved regions in BioEdit. After alignment, sequences were trimmed to specific lengths for each marker, and uncorrected pairwise distances (p-distances) were calculated using MEGA X software [58]. The resulting distance matrices are included in Supporting Information (S5–S6 Tables).

RESULTS

Taxonomic account of the new species

Phylum: Tardigrada Doyère 1840 [27]

Class: Eutardigrada Richters 1926 [59]

Order: Parachela Schuster et al. 1980 [11]

Superfamily: Macrobiotoidea Thulin 1928 [8]

Family: Macrobiotidae Thulin 1928 [8]

Genus: *Macrobiotus* Schultze 1834 [9]

Macrobiotus sharopovi sp. nov. Polishchuk, Kayastha, Młodzianowska, Warguła & Kaczmarek

(Figs 1–21, Tables 1–2)

Type Locality: Ecuador, Cotacachi-Cayapas National Park, Imbabura Province (00°19'47"N, 78°23'33"W; 3464 m asl), Zona de Intag; cryptograms on shrubs, 17th December 2014, leg. Milena Roszkowska and Łukasz Kaczmarek.

Material examined: In total, 177 specimens (148 animals and 29 eggs) were examined. The 90 animals and 19 eggs were mounted on microscope slides in Hoyer's medium, 38 animals and 10 eggs were fixed on SEM stubs and 20 animals were processed for DNA sequencing, out of which we found good quality barcodes from three specimens.

Type depositories: The holotype (1252-25) with 93 paratypes (79 animals and 14 eggs; slides: 1252-*, where the asterisk can be substituted by any of the following numbers: 1, 3, 4, 5, 9, 10, 14, 15, 16, 17, 18, 20, 22, 23, 24, 25, 26, 27 (animals) and 7, 8 (eggs)) are deposited at the Department of Animal Taxonomy and Ecology, Institute of Environmental Biology, Adam Mickiewicz University in Poznań, Uniwersytetu Poznańskiego 6, 61–614 Poznań, Poland. Ten paratypes and five eggs (slides: 1252-*, where the asterisk can be substituted by any of the following numbers: 6 (animals) and 28 (eggs)) are deposited in Institute of Systematics and Evolution of Animals, Polish Academy of Sciences, Sławkowska 17, 31-016, Kraków, Poland.

Etymology: The authors dedicated the species name to Ukrainian scientist Dr. Bizhan Sharopov, neurophysiologist, activist and soldier who tragically died in action defending Ukraine during the Russia–Ukraine War.

Animals (measurements and statistics in Table 1)

Body white to slightly yellowish in living specimens and transparent after fixation in Hoyer's medium. Eyes present in all fixed specimens (Fig 1). Entire cuticle covered with elliptical pores (0.8–1.8 µm in diameter) distributed throughout the body and clearly visible in both PCM and SEM (Figs 2–4). The edges of cuticular pores, in the SEM, evidently thicker compared with surrounding cuticle. Tiny granules inside pores absent. Easily visible granulation is present on legs I–IV (Figs 11–14, empty arrow).

Figs 1–4. *Macrobiotus sharopovi* sp. nov.: 1 – body, ventral view (holotype, PCM); 2 – body, dorsal view (paratype, SEM); 3–4 – cuticular pores on dorsal side of the body (paratype, PCM and SEM, respectively). Filled unindented arrowhead represents cuticular pores. Scale bars in µm.

Table 1. Measurements [in µm] and *pt* values of selected morphological structures of individuals of *Macrobiotus sharopovi* sp. nov., mounted in Hoyer's medium (N – number of specimens/structures measured; RANGE refers to the smallest and the largest structure among all measured specimens; SD – standard deviation, *pt* – ratio of the length of a given structure to the length of the buccal tube expressed as a percentage, ? – lack of measurements due to unsuitable position of the structure).

CHARACTER	N	RANGE						MEAN		SD		Holotype	
		µm			*pt*			µm	*pt*	µm	*pt*	µm	*pt*
Body length	20	333	–	492		–		397		45		418	
Buccopharyngeal tube													
Buccal tube length	20	29.7	–	38.7		–		34.0	–	2.1	–	34.0	–
Stylet support insertion point	20	21.6	–	28.4	*70.1*	–	*75.3*	25.1	*73.7*	1.5	*1.3*	25.6	*75.3*
Buccal tube external width	20	4.1	–	5.2	*11.9*	–	*15.4*	4.7	*13.7*	0.4	*0.8*	5.2	*15.4*
Buccal tube internal width	20	2.3	–	3.4	*7.0*	–	*9.9*	2.9	*8.4*	0.3	*0.6*	3.4	*9.9*
Ventral lamina length	15	17.0	–	23.0	*57.3*	–	*62.1*	20.5	*59.8*	1.5	*1.5*	20.8	*61.1*
Placoid lengths													
Macroplacoid 1	20	8.0	–	11.3	*25.3*	–	*31.2*	9.2	*27.0*	0.9	*1.5*	9.6	*28.3*
Macroplacoid 2	20	4.6	–	6.6	*14.2*	–	*18.4*	5.6	*16.6*	0.6	*1.1*	5.7	*16.8*
Microplacoid	20	2.0	–	3.0	*6.0*	–	*8.7*	2.5	*7.3*	0.2	*0.6*	3.0	*8.7*
Macroplacoid row	20	13.9	–	19.0	*45.1*	–	*51.9*	16.3	*47.8*	1.4	*2.0*	16.8	*49.3*
Placoid row	20	16.4	–	22.0	*54.0*	–	*60.5*	19.3	*56.7*	1.4	*1.9*	20.5	*60.2*
Claw I heights													
External primary branch	20	9.5	–	13.6	*29.4*	–	*35.6*	11.2	*32.9*	0.9	*1.9*	11.3	*33.4*

External secondary branch	20	7.2	–	10.1	*22.0*	–	*28.7*	8.5	*24.9*	0.8	*1.9*	9.8	*28.7*
Internal primary branch	20	8.8	–	12.5	*27.5*	–	*33.1*	10.3	*30.1*	0.8	*1.6*	10.5	*30.9*
Internal secondary branch	19	6.8	–	9.8	*20.6*	–	*26.6*	8.0	*23.5*	0.7	*1.5*	7.7	*22.7*
Claw II heights													
External primary branch	20	10.2	–	13.9	*31.9*	–	*38.0*	11.9	*35.1*	0.8	*1.9*	12.2	*36.0*
External secondary branch	20	7.9	–	10.4	*24.0*	–	*29.7*	9.2	*27.1*	0.7	*1.8*	10.0	*29.5*
Internal primary branch	20	8.7	–	13.0	*28.4*	–	*35.6*	11.0	*32.3*	0.9	*2.0*	11.2	*32.9*
Internal secondary branch	18	7.1	–	11.0	*21.6*	–	*28.3*	8.7	*25.5*	1.0	*2.0*	9.4	*27.7*
Claw III heights													
External primary branch	20	9.8	–	13.9	*32.1*	–	*38.7*	12.1	*35.6*	0.9	*2.1*	12.1	*35.7*
External secondary branch	18	7.5	–	12.3	*24.2*	–	*31.9*	9.2	*27.2*	1.1	*2.2*	8.5	*24.9*
Internal primary branch	20	9.3	–	13.3	*29.3*	–	*36.4*	11.1	*32.7*	0.8	*2.0*	10.8	*31.8*
Internal secondary branch	19	7.0	–	10.9	*23.5*	–	*29.3*	8.9	*26.1*	0.9	*1.9*	8.4	*24.8*
Claw IV lengths													
Anterior primary branch	18	9.8	–	15.4	*31.6*	–	*39.8*	12.2	*35.6*	1.2	*2.3*	12.6	*37.1*
Anterior secondary branch	17	7.7	–	11.0	*23.2*	–	*31.0*	9.4	*27.6*	1.0	*2.3*	9.8	*28.9*
Posterior primary branch	19	10.7	–	15.7	*34.9*	–	*41.9*	13.1	*38.5*	1.1	*1.9*	13.1	*38.4*
Posterior secondary branch	17	7.4	–	10.9	*24.0*	–	*32.4*	9.3	*27.5*	0.9	*2.5*	?	?

Bucco-pharyngeal apparatus of the *Macrobiotus* type, with ventral lamina and ten peribuccal lamellae. The OCA of the *patagonicus* type with second and third band of teeth visible with PCM (Figs 5–6), however, with SEM the first band of teeth is visible as well (Fig 7). The first band (visible only in SEM) consists of numerous extremely small teeth arranged in several rows situated anteriorly in the oral cavity behind the base of the peribuccal lamellae (Fig 7, empty unindented arrowhead). The second band is situated between the ring fold and the third band of teeth and composed of numerous small cones arranged into 3–4 rows (Figs 5–7, filled unindented arrowhead). The third band is located within the posterior portion of the oral cavity, between the second band of teeth and the buccal tube opening (Figs 5–6). This band of teeth is discontinuous and divided into a dorsal and ventral portion. The dorsal teeth form a single transverse ridge (Fig 5, filled indented arrowhead), whereas on the ventral side two separate lateral teeth are present (Fig 6, empty indented arrowhead).

Pharyngeal bulb spherical with triangular apophyses, two rod-shaped macroplacoids and a triangular microplacoid. Macroplacoid length sequence 2<1 (Figs 8–10). The first macroplacoid with central constriction (Figs 9–10, filled arrow and empty arrow). The second macroplacoid with sub-terminal constriction (Fig 10, filled black arrow).

Figs 5–10. *Macrobiotus sharopovi* sp. nov.: 5 – oral cavity armature, dorsal view (holotype, PCM); 6 – oral cavity armature, ventral view (holotype, PCM); 7 – oral cavity armature (paratype, SEM); 8 – bucco-pharyngeal apparatus, dorsal view (holotype, PCM); 9 – placoid row, dorsal view (holotype, PCM); 10 – placoid row, ventral view (holotype, PCM). Empty unindented arrowhead represents first band of teeth, filled unindented arrowhead represents second band of teeth, filled indented arrowhead represents third band of teeth on the dorsal view, empty indented arrowhead represents third band of teeth on the ventral view, filled arrow represents first macroplacoid with central constriction on dorsal side, empty arrow represents first macroplacoid with central constriction on ventral side and filled black arrow represents second macroplacoid with sub-terminal constriction. Scale bars in μm.

Claws Y-shaped of the *hufelandi* type, stout (Figs 11–14). Under claws I–III, cuticular bars (Fig 11, filled indented arrowhead), and doubled muscle attachments (Figs 11–12, empty indented arrowhead) are present. Primary branches with distinct accessory points (Figs 11–14, filled arrow) and a stalk connecting lunules and claws. Lunules under claws I–III smooth (Figs 11–12, filled unindented arrowhead) and dentated under claws IV (Figs 13–14, empty unindented arrowhead).

Figs 11–14. *Macrobiotus sharopovi* sp. nov.: 11 – claws III (paratype, PCM); 12 – claws II (paratype, SEM); 13 – claws IV (paratype, PCM); 14 – claws IV (paratype, SEM). Filled arrow represents accessory points on primary branches of claws, empty arrow represents granulations present on legs, filled unindented arrowhead represents smooth lunules, empty unindented arrowhead represents dentated lunules, filled indented arrowhead represents cuticular bar and empty indented arrowhead represents double muscle attachments. Scale bars in μm.

Eggs (measurements and statistics in Table 2)

Spherical, white, ornamented and laid freely (Figs 15–16) with *hufelandi* type egg chorion ornamentation (Figs 17–18). The surface between processes reticulated with mesh of

0.6–1.1 µm in diameter. Egg processes in shape of concave cones with concave and indented smooth terminal discs and few long flexible smooth filaments on them (Figs 18–21).

Figs 15–21. *Macrobiotus sharopovi* sp. nov.: 15–16 – egg chorion (paratype, PCM and SEM, respectively); 17–18 – the surface between egg processes (paratype, PCM and SEM, respectively); 19 – egg circumference with egg processes midsections (paratype, PCM); 20–21 – egg processes (paratype, SEM). Filled unindented arrowhead represents flexible filaments on the egg processes. Scale bars in µm.

Table 2. Measurements [in µm] of selected morphological structures of eggs of *Macrobiotus sharopovi* sp. nov. mounted in Hoyer's medium (N – number of specimens/structures measured, RANGE refers to the smallest and the largest structure among all measured eggs; SD – standard deviation).

CHARACTER	N	RANGE			MEAN	SD
Egg bare diameter	17	70.0	–	84.1	75.6	5.1
Egg full diameter	17	81.7	–	97.9	87.1	4.8
Process height	51	4.2	–	6.3	5.2	0.6
Process base width	51	4.4	–	6.8	5.6	0.6
Process base/height ratio	51	101%	–	121%	108%	5%
Terminal disc width	51	3.5	–	5.5	4.5	0.5
Inter-process distance	51	2.4	–	4.9	3.9	0.6
Number of processes on the egg circumference	15	20	–	24	21.6	1.5

Remarks

Following normalization using Thorpe's method (see S1 Table), 14 of the 26 morphological traits examined showed statistical significance. Traits such as body length, macroplacoid 1, claw II internal primary branch, claw III external and internal secondary branch, claw IV anterior and posterior primary branch, claw IV anterior secondary branch, exhibited an allometric relationship characterized by a growth rate disproportionate to overall body size, as indicated by 'b' values significantly greater than 1. Conversely, the buccal tube external width, claw I external primary branch, claw external primary and secondary branches, claw III external and internal primary branch and claw IV posterior secondary branches

displayed an allometric relationship with growth rates that are slower relative to body size, evidenced by 'b' values significantly less than 1.

DNA sequences

We obtained good quality sequences for the following molecular markers:

- 18S rRNA: GenBank: XXXXX; 1260bp long; (species voucher number: 1252.11).
- 28S rRNA: GenBank: XXXXX; 777bp long; (species voucher number: 1252.14).
- COI: GenBank: XXXXX; 662bp long; (species voucher number: 1252.12).
- ITS-2: GenBank: XXXXX–XX; 379bp long; (species voucher numbers: 1252.11 and 1252.14).

Genetic distances

The ranges of uncorrected genetic p-distances between the molecular markers of *Mac. sharopovi* sp. nov. obtained in our study and the sequences of all species of the genus *Macrobiotus* available in GenBank are as follows (S5 Table):

- 18S rRNA: 0.2%–2.6% (1.6% on average), with the most similar being *Mac. shonaicus* Stec, Arakawa & Michalczyk 2018 (GenBank: MG757132) [60], and the least similar being *Mac. naginae* Vecchi, Stec, Vouri, Ryndov, Chartrain & Calhim 2022 (GenBank: OK663219–20) [61].
- 28S rRNA: 2.6%–10.1% (6.3% on average), with the most similar being *Mac. polypiformis* Roszkowka, Ostrowska, Stec, Janko & Kaczmarek 2017 (GenBank: KX810009) [39] and the least similar being *Mac. naginae* Vecchi, Stec, Vouri, Ryndov, Chartrain, Calhim & 2022 (GenBank: OK663230–31) [61].

- COI: 18.4%–30.5% (22.5% on average), with the most similar being *Mac. paulinae* Stec, Smolak, Kaczmarek & Michalczyk 2015 (GenBank: KT951668) [42], and the least similar being *Mac. rebecchi* Stec 2022 (GenBank: OP477442–43) [24].
- ITS2 rRNA: 15.9%–30.4 % (23.6% on average), with the most similar being *Mac. papei* Stec, Kristensen & Michalczyk 2018 (GenBank: MH063921) [62] and the least similar being *Mac. vladimiri* Bertolani, Biserov, Rebecchi & Cesari 2011 (GenBank: MN888347) [21].

Morphological differential diagnosis

The new species based on the morphology of eggs belongs to the *Mac. paulinae* morpho-group according to classification in Kaczmarek et al. [16] and it is most similar to *Mac. papei*, *Mac. paulinae*, *Mac. polypiformis* and *Mac. shonaicus*. But it differs specifically from the following:

1. ***Macrobiotus papei***, reported only from the type locality in Tanzania [62], by: larger pores/mesh on the egg chorion (0.6–1.1 µm in *Mac. sharopovi* sp. nov. *vs* 0.3–0.6 µm in *Mac. papei*), smaller number of filaments on the egg processes discs and smaller number of processes on the egg circumference (20–24 in *Mac. sharopovi* sp. nov. *vs* 26–32 in *Mac. papei*).
2. ***Macrobiotus paulinae***, reported only from the type locality in Kenya [33,42], by: the absence of dorso-lateral patches of granulation on the cuticle, larger diameter of cuticular pores (0.8–1.8 µm in *Mac. sharopovi* sp. nov. *vs* 0.3–0.5 µm in *Mac. paulinae*), presence of smooth filaments on the egg processes and larger pores/mesh on the egg chorion (0.6–1.1 µm in *Mac. sharopovi* sp. nov. *vs* 0.05–0.2 µm in *Mac. paulinae*).
3. ***Macrobiotus polypiformis***, reported only from the type locality in Ecuador [39], by: higher *pt* of the ventral lamina (*pt=57.3–62.1* in *Mac. sharopovi* sp. nov. *vs pt=52.1–*

55.1 in *Mac. polypiformis*), longer macroplaciod row and placoid row (13.9–19.0 µm [*pt=45.1–51.9*] and 16.4–22.0 µm [*pt=54.0–60.5*], respectively in *Mac. sharopovi* sp. nov. *vs* 9.0–11.8 µm [*pt=34.3–39.9*] and 11.1–14.5 µm [*pt=41.4–49.0*], respectively in *Mac. polypiformis*), presence of smooth filaments on egg processes discs and by smaller number of these filaments.

4. ***Macrobiotus shonaicus***, reported only from Japan [60,63], by: the absence of the cuticular bulge resembling pulvinus like structure and faint cuticular fold on legs I–III, larger diameter of cuticular pores (0.8–1.8 µm in *Mac. sharopovi* sp. nov. *vs* 0.2–0.4 µm in *Mac. shonaicus*), the presence of the reticulation on the egg surface between egg processes, smaller number of the egg processes on the egg circumference (20–24 in *Mac. sharopovi* sp. nov. *vs* 28–36 in *Mac. shonaicus*), presence of smooth filaments on egg processes discs and by smaller number of these filaments.

Genus: *Mesobiotus* (Vecchi, Cesari, Bertolani, Jönsson & Guidetti 2016) [10]

Mesobiotus shevchenkoi sp. nov. Polishchuk, Kayastha, Michalska, Warguła, & Kaczmarek

(Figs 22–41, Tables 3–4)

Type Locality: Ecuador, Cotacachi-Cayapas National Park, Imbabura Province (00°17'31"N, 78°21'26"W; 3093 m asl); moss on rock, 16th December 2014, leg. Milena Roszkowska and Łukasz Kaczmarek.

Material examined: In total 127 specimens (87 animals and 40 eggs) were examined. The 55 animals and 30 eggs were mounted on microscope slides in Hoyer's medium, 25 animals and 10 eggs were fixed on SEM stubs and seven animals were processed for DNA sequencing, out of which we found good quality barcodes from one specimen.

Type depositories: The holotype (1211-2) with 61 paratypes (34 animals and 27 eggs; slides: 1211-*, where the asterisk can be substituted by any of the following numbers: 1, 2, 3, 4, 5, 6, 10, 11, 12, 16, 17 (animals) and 7, 8, 13, 14, 15 (eggs)) are deposited in the Department of Animal Taxonomy and Ecology, Institute of Environmental Biology, Adam Mickiewicz University, Poznań, Uniwersytetu Poznańskiego 6, 61-614 Poznań, Poland. Twenty paratypes and three eggs (slide: 1252-*, where the asterisk can be substituted by any of the following numbers: 18 (animals) and 9 (eggs)) are deposited in Institute of Systematics and Evolution of Animals, Polish Academy of Sciences, Sławkowska 17, 31-016, Kraków, Poland.

Etymology: The authors dedicated the species name to the great Ukrainian poet, writer, artist, folklorist and ethnographer Taras Shevchenko (1814–1861), who remains a key figure to Ukrainian culture.

Animals (all measurements and statistics in Table 3)

Live specimens white, but after fixation in Hoyer's medium transparent. Eyes present in all fixed specimens (Fig 22). Dorsal, lateral and ventral cuticle smooth and without pores. Granulation on legs I–IV present and well visible in PCM (Figs 23–26, empty unindented arrowhead). Thin singular cuticular bars (Fig 23, filled indented arrowhead) and double muscle attachments under claws I-III present (Figs 23–24, empty indented arrowhead).

Table 3. Measurements [in µm] and *pt* values of selected morphological structures of individuals of *Mesobiotus shevchenkoi* sp. nov., mounted in Hoyer's medium (N – number of specimens/structures measured; RANGE refers to the smallest and the largest structure among all measured specimens; SD – standard deviation, *pt* – ratio of the length of a given structure to the length of the buccal tube expressed as a percentage, ? – lack of measurements due to unsuitable position of the structure).

CHARACTER	N	RANGE				MEAN		SD		Holotype	
		µm			*pt*	µm	*pt*	µm	*pt*	µm	*pt*
Body length	20	329	–	459	–	392		38		393	

Buccopharyngeal tube													
Buccal tube length	20	30.6	–	39.7		–		36.6	–	2.5	–	37.7	–
Stylet support insertion point	20	23.1	–	30.9	*74.5*	–	*78.7*	28.0	*76.6*	2.0	*1.1*	28.5	*75.5*
Buccal tube external width	20	4.4	–	7.0	*13.9*	–	*17.7*	5.7	*15.5*	0.7	*1.1*	5.9	*15.6*
Buccal tube internal width	20	3.3	–	5.5	*9.6*	–	*14.1*	4.2	*11.5*	0.6	*1.2*	4.5	*11.8*
Ventral lamina length	17	18.1	–	24.6	*58.1*	–	*62.9*	22.3	*61.0*	1.7	*1.3*	23.4	*62.0*
Placoid lengths													
Macroplacoid 1	20	4.5	–	7.1	*13.6*	–	*17.9*	5.7	*15.6*	0.7	*1.3*	5.3	*14.1*
Macroplacoid 2	20	3.6	–	5.8	*9.9*	–	*14.7*	4.5	*12.1*	0.6	*1.1*	3.7	*9.9*
Macroplacoid 3	20	4.3	–	6.9	*13.0*	–	*17.3*	5.3	*14.5*	0.7	*1.2*	5.1	*13.6*
Microplacoid	20	2.6	–	3.8	*7.5*	–	*10.5*	3.1	*8.6*	0.3	*0.7*	3.0	*7.9*
Macroplacoid row	20	14.4	–	22.6	*46.0*	–	*57.1*	18.1	*49.3*	2.2	*3.3*	17.3	*46.0*
Placoid row	20	17.8	–	28.0	*57.2*	–	*70.8*	22.3	*60.9*	2.5	*3.5*	21.7	*57.5*
Claw I heights													
External primary branch	18	8.8	–	11.5	*25.4*	–	*30.7*	10.2	*27.9*	0.9	*1.5*	10.1	*26.9*
External secondary branch	18	6.6	–	8.7	*18.0*	–	*23.2*	7.7	*21.0*	0.7	*1.7*	8.2	*21.7*
Internal primary branch	18	8.6	–	11.3	*24.2*	–	*28.6*	9.7	*26.5*	0.7	*1.3*	?	?
Internal secondary branch	18	5.9	–	8.0	*16.4*	–	*22.8*	7.1	*19.4*	0.7	*1.5*	?	?
Claw II heights													
External primary branch	20	9.2	–	11.9	*26.9*	–	*31.4*	10.6	*29.1*	0.7	*1.3*	10.6	*28.2*
External secondary branch	20	6.3	–	8.9	*17.4*	–	*23.8*	7.8	*21.2*	0.7	*1.6*	7.4	*19.6*
Internal primary branch	19	8.3	–	11.2	*25.7*	–	*30.8*	10.1	*27.7*	0.8	*1.6*	9.7	*25.7*
Internal secondary branch	17	6.4	–	8.6	*17.1*	–	*24.0*	7.7	*21.0*	0.7	*1.7*	?	?
Claw III heights													
External primary branch	19	9.1	–	11.9	*26.6*	–	*32.3*	10.7	*29.3*	0.8	*1.7*	11.0	*29.2*
External secondary branch	18	7.0	–	8.9	*18.3*	–	*25.8*	7.9	*21.7*	0.6	*2.0*	7.0	*18.6*
Internal primary branch	19	8.9	–	11.5	*24.4*	–	*32.2*	10.3	*28.3*	0.7	*1.9*	10.5	*27.8*
Internal secondary branch	18	6.2	–	8.6	*18.0*	–	*23.9*	7.5	*20.6*	0.6	*1.8*	7.5	*19.8*
Claw IV lengths													
Anterior primary branch	19	10.5	–	13.7	*29.5*	–	*36.4*	11.8	*32.5*	0.9	*1.9*	12.1	*32.2*
Anterior secondary branch	19	7.3	–	10.4	*21.2*	–	*26.7*	8.6	*23.6*	0.7	*1.5*	8.7	*23.1*
Posterior primary branch	20	10.8	–	14.3	*30.6*	–	*38.4*	12.4	*34.0*	1.0	*2.1*	13.4	*35.6*
Posterior secondary branch	20	7.6	–	10.2	*21.3*	–	*26.3*	8.8	*24.0*	0.7	*1.6*	8.7	*23.1*

Bucco-pharyngeal apparatus of *Mesobiotus* type with ten peribuccal lamellae and the ventral lamina. The OCA of the *harmsworthi* type with three bands of teeth, visible in PCM (Figs 27–29). The first band is composed of small teeth that are clearly visible in PCM as granules (Figs 28–30, filled indented arrowhead). The second band is composed of teeth that are arranged in a row parallel to the main axis of the buccal tube as comma-shaped ridges (Figs 27, 29, filled unindented arrowhead). The third band is composed of teeth that are arranged as a system of three dorsal and three ventral transverse ridges (Figs 27–29, 31, empty unindented arrowhead). The central ventral ridge is almost round and shorter, resembling a single tooth (Fig 28) (sometimes divided into two smaller teeth as on Fig 29), while the lateral ventral ridges

are curved, longer, and thicker than the middle one, but they are thinner at the external extremities (Figs 28–29). Pharyngeal bulb round with apophyses, three rod-shaped macroplacoids and large microplacoid situated in close proximity to the final macroplacoid (Fig 32). Macroplacoid lengths sequence is $2<3<1$. Both the first and the second macroplacoids are widened at the end, and the first macroplacoid is constricted. (Figs 33–34, filled indented arrowhead and empty indented arrowhead). The third macroplacoid lacks a terminal protrusion, but has a shallow, conspicuous subterminal constriction (Figs 33–34, filled unindented arrowhead and empty unindented arrowhead).

Claws of the *Mesobiotus* type with a short common tract with septa at its proximal part (Figs 23–26). All primary branches with accessory points (Figs 23–26, filled arrow). External and posterior claws longer than internal and anterior claws. Claws on leg IV larger than claws on other legs. Lunules under all claws smooth (Figs 23–26, filled unindented arrowhead).

Figs 22–26. *Mesobiotus shevchenkoi* sp. nov.: 22 – body, ventral view (holotype, PCM); 23 – claws III (holotype, PCM); 24 – claws II (paratype, SEM); 25 – claws IV (holotype, PCM); 26 – claws IV (paratype, SEM). Filled arrow represents accessory points on primary branches of claws, filled unindented arrowhead represents smooth lunulas, empty unindented arrowhead represents granulations present on legs, filled indented arrowhead represents cuticular bar and empty indented arrowhead represents double muscle attachments. Scale bars in µm.

Figs 27–31. *Mesobiotus shevchenkoi* sp. nov.: 27 – oral cavity armature, dorsal view (paratype, PCM); 28 – oral cavity armature, ventral view (holotype, PCM); 29 – oral cavity armature, ventral view (paratype, PCM); 30–31 – oral cavity armature (paratype, SEM). Filled indented arrowhead represents the first band of teeth, filled unindented arrowhead represents the second band of teeth and empty unindented arrowhead represents the third band of teeth. Scale bars in µm.

Figs 32–34. *Mesobiotus shevchenkoi* sp. nov.: 32 – bucco-pharyngeal apparatus, dorsal view (holotype); 33 – placoid row, dorsal view (holotype); 34 – placoid row, ventral view (holotype). Filled indented arrowhead and

empty indented arrowhead represent central constriction in first macroplacoid on the dorsal side and on the ventral side respectively, filled unindented arrowhead and empty unindented arrowhead represent subterminal constriction on the third macroplacoid on the dorsal side and on the ventral side respectively. All PCM. Scale bars in µm.

Eggs (all measurements and statistics in Table 4)

Spherical, white and laid freely (Figs 35–36). Egg chorion with processes in the shape of wide sharp cones with thick, but flexible, short smooth endings. Eggshell covered with small dots and wrinkles (Fig 37, in PCM), which in SEM are visible as wrinkles of diverse shapes (Fig 38). Egg processes bases are surrounded by a crown of slightly elongated thickening (Figs 37–38), and in SEM surface of processes is smooth or rarely with small pores on processes bases. The labyrinthine layer is visible under PCM as a reticulum in the process walls, with varying mesh size uniformly distributed within the process walls (Figs 37, 39–40). In SEM, instead, egg processes covered by shallow hollows, distributed regularly (Figs 38, 41). Apical parts of some processes are bifurcated or divided into even shorter filaments (Figs 39–41).

Figs 35–41. *Mesobiotus shevchenkoi* sp. nov.: 35–36 – egg chorion (paratype, PCM and SEM, respectively); 37–38 – the surface between egg processes (paratype, PCM and SEM, respectively); 39–40 – egg processes midsections (paratype, PCM); 41 – egg processes (paratype, SEM). Scale bars in µm.

Table 4. Measurements [in µm] of selected morphological structures of eggs of *Mesobiotus shevchenkoi* sp. nov. mounted in Hoyer's medium (N – number of specimens/structures measured, RANGE refers to the smallest and the largest structure among all measured eggs; SD – standard deviation).

CHARACTER	N	RANGE			MEAN	SD
Egg bare diameter	11	61.9	–	72.9	66.7	4.5
Egg full diameter	10	80.7	–	93.7	86.8	4.9
Process height	26	10.6	–	13.7	12.0	0.9
Process base width	26	10.1	–	14.0	12.2	1.1
Process base/height ratio	26	78%	–	119%	102%	12%
Inter-process distance	24	2.5	–	3.8	3.0	0.3

Number of processes on the egg circumference	4	11	–	14	12.5	1.3

Remarks

Following normalization using Thorpe's method (see S2 Table), 22 of the 28 morphological traits examined showed statistical significance. Traits such as buccal tube external width, buccal tube internal width, and macroplacoids 1, 2, and 3, along with the macroplacoid row, exhibited an allometric relationship characterized by a growth rate disproportionate to overall body size, as indicated by 'b' values significantly greater than 1. Conversely, the microplacoid and the claws (including the external secondary and internal branches of claw I, as well as claws II, III, and IV) displayed an allometric relationship with growth rates that are slower relative to body size, evidenced by 'b' values significantly less than 1.

DNA sequences

We obtained good quality sequences for the following molecular markers:

- 28S rRNA: GenBank: XXXXX–XX; 746 bp long (species voucher numbers: 1211.5).
- COI: GenBank: XXXXX–XX; 624 bp long (species voucher numbers: 1211.5).
- ITS-2: GenBank: XXXXX–XX; 362 bp long (species voucher numbers: 1211.5).

Genetic distances

The ranges of uncorrected genetic p-distances between the molecular markers of *Meb. shevchenkoi* sp. nov. obtained in our study and the sequences of all species of the genus *Mesobiotus* available in GenBank are as follows (S6 Table):

- 28S rRNA: 6.1%–13.7% (8.1% on average), with the most similar being *Meb. vulpinus* Tumanov, Androsova, Gavrilenko & Kalimullin 2024 (GenBank: OR805140–41) [22],

and the least similar being *Meb. dilimanensis* Itang, Stec, Mapalo, Mirano-Bascos & Michalczyk 2020 (GenBank: MN257049) [64].

- COI: 19.3%–27.6% (22.6% on average), with the most similar being *Meb. harmsworthi* Murray 1907 [65] (GenBank: MN313170) [66], and the least similar being *Meb.* cf. *furciger* (GenBank: MW727958) [23].
- ITS2 rRNA: 13.6%–43.4% (23.2% on average), with the most similar being *Meb. occultatus* Kaczmarek, Zawierucha, Buda, Stec, Gawlak, Michalczyk & Roszkowska 2018 (GenBank: MH197155) [66], and the least similar being *Meb. marmoreus* Stec 2021 (GenBank: OL257861–63) [67].

Morphological differential diagnosis

The new species belongs to the *Meb. harmsworthi* morpho-group. By having eggshell surface with dots and/or wrinkles and egg processes in the shape of wide sharp cones with reticulated surface and surrounded by crown of thickenings or/and wrinkles on bases, the new species is similar to the following taxa: *Meb. coronatus* (De Barros 1942) [68], *Meb. insuetus* (Pilato, Sabella & Lisi 2014) [69], *Meb. occultatus, Meb. patiens* (Pilato, Binda, Napolitano & Moncada 2000) [70], *Meb. philippinicus* Mapalo, Stec, Mirano-Bascos & Michalczyk 2016 [71], *Meb. pseudocoronatus* (Pilato, Binda & Lisi 2006) [72], *Meb. simulans* (Pilato, Binda, Napolitano & Moncada 2000) [70]. But it differs specifically from:

1. ***Mesobiotus coronatus***, reported from many localities around the globe [31,73], but with confirmed reports only in South America [31,68,70], by: larger egg bare and full diameter (61.9–72.9 µm and 80.7–93.7 µm, respectively in *Meb. shevchenkoi* sp. nov. *vs* 42.0–55.0 µm and 55.0–71.0 µm, respectively in *Meb. coronatus*) and longer egg processes (10.6–13.7 µm in *Meb. shevchenkoi* sp. nov. vs *ca* 9.2 µm in *Meb. coronatus*).

2. ***Mesobiotus insuetus***, reported only from the type locality in Italy [69], by: the presence of eyes, typically developed claws IV (on posterior and anterior claws primary and secondary branches are joined for longer basal tract and forming a right angle in *Meb. insuetus*) and longer egg processes (10.6–13.7 µm in *Meb. shevchenkoi* sp. nov. *vs ca* 7.9–8.6 µm in *Meb. insuetus*).
3. ***Mesobiotus occultatus***, reported only from the type locality in Svalbard Archipelago, Norway [66], by: a bit different macroplacoid length sequence ($2<3<1$ in *Meb. shevchenkoi* sp. nov. *vs* $2<3\leq1$ in *Meb. occultatus*), larger *pt* of the anterior primary branch and posterior primary branch of the claws IV (*pt=29.5–36.4* and *pt=30.6–38.4* respectively in *Meb. shevchenkoi* sp. nov. *vs pt=24.6–29.4* and *pt=25.6–29.7* in *Meb. occultatus*), smaller egg full diameter (80.7–93.7 µm in *Meb. shevchenkoi* sp. nov. *vs* 97.4–126.6 µm in the *Meb. occultatus*), shorter egg processes (10.6–13.7 µm in *Meb. shevchenkoi* sp. nov. *vs* 14.1–21.8 µm in the *Meb. occultatus*) and the absence of granulation at the processes' apical parts.
4. ***Mesobiotus patiens***, reported only from type locality in Italy and few localities in Germany [70,74], by: the presence of eyes and smaller egg bare diameter (61.9–72.9 µm in *Meb. shevchenkoi* sp. nov. *vs ca.* 75.0–87.0 µm in the *Meb. patiens*).
5. ***Mesobiotus philippinicus***, reported only from the type locality in Philippines [71], by: the absence of dentated lunules under claws IV, smaller number of the egg processes on the egg circumference (11–14 in *Meb. shevchenkoi* sp. nov. *vs* 15–17 in *Meb. philippinicus*) and the absence of granulation at the processes' apical parts.
6. ***Mesobiotus pseudocoronatus***, reported only from the type locality in Seychelles [72], by: smooth cuticle (cuticle with small dorsal and lateral tubercles in *Meb. pseudocoronatus*), the absence of dentated lunules under claws IV and larger egg bare

diameter (61.9–72.9 µm in *Meb. shevchenkoi* sp. nov. *vs ca.* 50.1 µm in *Meb. pseudocoronatus*).

7. ***Mesobiotus simulans***, reported from few localities in Italy [70], by: the absence of dentated lunules under claws IV.

Superfamily: Hypsibioidea Pilato 1969 [75] (in Marley et al. [76])

Family: Ramazzottiidae Sands, McInnes, Marley, Goodall-Copestake, Convey & Linse 2008 [25]

Genus: *Ramazzottius* Binda & Pilato 1986 [26]

Ramazzottius syraxi sp. nov. Polishchuk, Kayastha, Warguła & Kaczmarek

(Figs 42–53, Tables 5–6)

Type locality: Ecuador, Cotacachi-Cayapas National Park, Imbabura Province (00°17'46"N, 78°20'52"W; 3122 m asl), near Lago Cotacachi; mosses and lichens on shrubs, 16th December 2014, leg. Łukasz Kaczmarek and Milena Roszkowska.

Additional locality: Ecuador, Cotacachi-Cayapas National Park, Imbabura Province (00°18'02"N, 78°20'47"W; 3176 m asl), cryptograms on shrubs, 16th December 2014, leg. Łukasz Kaczmarek and Milena Roszkowska.

Material examined: In total 13 specimens were examined (seven specimens from the type locality and six specimens from the additional locality) and mounted on microscope slides in Hoyer's medium.

Type depositories: The holotype (1219-13) and six paratypes (slides: 1219-*, where the asterisk can be substituted by any of the following numbers: 9, 22), as well as three microscope slides (1132-2, 1132-3, 1132-4) with six specimens from the additional locality are deposited in the Department of Animal Taxonomy and Ecology, Institute of Environmental Biology,

Adam Mickiewicz University, Poznań, Uniwersytetu Poznańskiego 6, 61-614 Poznań, Poland. Two paratypes from the type locality (slide 1219-4) are deposited in Institute of Systematics and Evolution of Animals, Polish Academy of Sciences, Sławkowska 17, 31-016, Kraków, Poland.

Etymology: The authors dedicated the species name to the fantasy world "A Song of Ice and Fire" by George R.R. Martin. The species is named after the dragon Syrax belonged to the book character Rhaenyra Targaryen (from the book "Fire and Blood") due to the visual similarity caused by the cuticle appearance of *Ram. syraxi* sp. nov.

Animals (all measurements and statistics in Tables 5–6)

Body colour yellow-orange in living specimens. Eyes absent in live animals. A pair of elliptical organs present on the head. Dorsal cuticle with strong sculpture and less visible gibbosities (Figs 42, 44, Figs 45–48), while the ventral cuticle is smooth (Fig 43). Dorsal side of the body with eight rows of gibbosities (configuration: VIII:1-2-3-4-5-4-3-1) (Figs 42, 44). Gibbosities and cuticle between them covered by smaller and larger polygons (0.8–3.8 µm in diameter) sometimes composed of few smaller granules (Figs 42, 44; Figs 45–48). In rows II, IV and VI a concave areas, less strongly sculptured, are present between laterally situated gibbosities. In rows I and VIII, a wide and convex sculptured stripes are present which are considered as single gibbosities. The appearance of polygons on gibbosities and cuticle between them may vary slightly in different individuals (Figs 45–48). Cuticle on all legs smooth.

Figs 42–44. *Ramazzottius syraxi* sp. nov.: 42 – body, dorsal view (holotype); 43 – body, ventral view (holotype); 44 – body, dorso-ventral view (paratype). All PCM. Scale bars in µm.

Figs 45–48. *Ramazzottius syraxi* sp. nov.: 45 – cuticle, dorsal view, rows III-IV-V-VI (holotype); 46 – cuticle, dorsal view, rows III-IV-V-VI (paratype); 47 – cuticle, dorsal view (paratype); 48 – cuticle, dorsal view, rows III-IV-V-VI (paratype). All PCM. Scale bars in µm.

Table 5. Measurements [in µm] and *pt* values of selected morphological structures of individuals of *Ramazzottius syraxi* sp. nov. from the type locality, mounted in Hoyer's medium (N – number of specimens/structures measured; RANGE refers to the smallest and the largest structure among all measured specimens; SD – standard deviation, *pt* – ratio of the length of a given structure to the length of the buccal tube expressed as a percentage, ? – lack of measurements due to unsuitable position of the structure).

**CHARACTER**	**N**	**RANGE**		**MEAN**		**SD**		**Holotype**	
		**µm**	***pt***	**µm**	***pt***	**µm**	***pt***	**µm**	***pt***
Body length	5	201 – 416	–	323		91		349	
Buccopharyngeal tube									
Buccal tube length	5	25.1 – 36.5	–	31.8	–	4.4	–	31.6	–
Stylet support insertion point	5	14.3 – 20.7	*56.6 – 59.4*	18.5	*58.3*	2.6	*1.4*	18.8	*59.4*
Buccal tube external width	5	2.1 – 2.8	*7.8 – 8.6*	2.6	*8.2*	0.3	*0.4*	2.7	*8.5*
Buccal tube internal width	5	0.8 – 1.2	*2.6 – 3.8*	1.0	*3.3*	0.2	*0.4*	0.8	*2.6*
Placoid lengths									
Macroplacoid 1	5	2.7 – 3.5	*8.4 – 10.9*	3.1	*9.8*	0.4	*0.9*	2.7	*8.4*
Macroplacoid 2	5	2.3 – 3.2	*8.3 – 9.2*	2.8	*8.7*	0.4	*0.4*	2.7	*8.6*
Macroplacoid row	5	5.4 – 7.9	*20.4 – 22.5*	6.8	*21.3*	1.0	*0.8*	6.5	*20.4*
Claw I heights									
External base	5	4.2 – 10.2	*16.9 – 28.9*	8.0	*24.7*	2.3	*4.6*	8.4	*26.7*
External primary branch	5	7.3 – 12.7	*28.9 – 36.1*	10.6	*33.2*	2.1	*2.6*	10.8	*34.0*
External secondary branch	4	5.7 – 8.9	*18.7 – 24.5*	7.3	*21.6*	1.3	*2.5*	7.1	*22.6*
Internal base	5	4.3 – 8.2	*16.7 – 22.4*	6.3	*19.5*	1.6	*2.6*	5.3	*16.7*
Internal primary branch	4	5.6 – 9.3	*21.0 – 26.5*	7.1	*23.1*	1.6	*2.4*	6.6	*21.0*
Internal secondary branch	5	5.1 – 7.6	*16.3 – 21.7*	6.2	*19.5*	0.9	*2.1*	6.5	*20.6*
Claw II heights									
External base	5	7.2 – 10.8	*28.7 – 30.7*	9.4	*29.6*	1.5	*0.8*	9.5	*30.1*
External primary branch	5	8.2 – 13.2	*32.5 – 36.2*	11.0	*34.3*	2.0	*1.7*	11.2	*35.5*
External secondary branch	5	5.5 – 9.4	*18.1 – 27.4*	7.7	*23.9*	2.0	*4.0*	8.7	*27.4*
Internal base	5	5.1 – 8.4	*19.9 – 23.8*	6.8	*21.3*	1.4	*1.7*	6.3	*20.0*
Internal primary branch	3	6.0 – 9.5	*23.7 – 27.2*	7.7	*24.9*	1.8	*1.9*	7.5	*23.7*
Internal secondary branch	5	4.5 – 8.1	*18.1 – 23.1*	6.7	*21.0*	1.4	*2.0*	7.1	*22.3*
Claw III heights									
External base	5	7.1 – 10.7	*24.0 – 30.5*	8.7	*27.4*	1.4	*2.6*	9.1	*28.8*
External primary branch	5	8.1 – 14.0	*29.5 – 40.0*	11.2	*34.9*	2.5	*4.1*	11.9	*37.7*
External secondary branch	5	5.2 – 9.8	*20.6 – 27.9*	7.6	*23.8*	1.8	*2.9*	8.0	*25.4*
Internal base	5	4.9 – 7.8	*17.3 – 22.6*	6.5	*20.2*	1.3	*2.2*	7.2	*22.6*
Internal primary branch	2	5.6 – 8.7	*22.2 – 24.8*	7.1	*23.5*	2.2	*1.9*	?	?
Internal secondary branch	5	4.7 – 8.0	*15.4 – 22.9*	6.2	*19.4*	1.3	*2.9*	5.9	*18.7*
Claw IV lengths									
Anterior base	5	4.1 – 6.8	*16.2 – 20.2*	5.9	*18.4*	1.1	*1.6*	6.4	*20.2*
Anterior primary branch	5	5.8 – 9.0	*22.9 – 24.8*	7.5	*23.7*	1.3	*0.8*	7.3	*22.9*

Anterior secondary branch	4	4.0 – 7.6	*15.8* – *21.7*	6.1	*19.0*	1.5	*3.1*	6.9	*21.7*
Posterior base	5	6.1 – 10.5	*22.9* – *29.9*	8.4	*26.3*	1.5	*2.8*	8.4	*26.5*
Posterior primary branch	5	8.7 – 17.3	*34.8* – *49.3*	13.9	*43.2*	3.5	*5.5*	13.7	*43.2*
Posterior secondary branch	2	7.0 – 7.3	*19.1* – *23.0*	7.1	*21.0*	0.2	*2.8*	7.3	*23.0*

Table 6. Measurements [in µm] and *pt* values of selected morphological structures of individuals of *Ramazzottius syraxi* sp. nov. from the additional locality, mounted in Hoyer's medium (N – number of specimens/structures measured; RANGE refers to the smallest and the largest structure among all measured specimens; SD – standard deviation, *pt* – ratio of the length of a given structure to the length of the buccal tube expressed as a percentage, ? – lack of measurements due to unsuitable position of the structure).

CHARACTER	N	RANGE		MEAN		SD	
		µm	*pt*	µm	*pt*	µm	*pt*
Body length	4	137 – 274	–	226		64	
Buccopharyngeal tube							
Buccal tube length	4	22.1 – 29.4	–	26.4	–	3.1	–
Stylet support insertion point	4	13.2 – 17.2	*57.3* – *59.8*	15.5	*58.7*	1.7	*1.1*
Buccal tube external width	4	1.8 – 2.2	*7.3* – *8.3*	2.0	*7.7*	0.2	*0.4*
Buccal tube internal width	4	0.8 – 0.9	*2.9* – *3.8*	0.8	*3.2*	0.0	*0.4*
Placoid lengths							
Macroplacoid 1	4	2.1 – 3.0	*9.3* – *10.7*	2.7	*10.0*	0.4	*0.6*
Macroplacoid 2	4	1.8 – 2.6	*8.1* – *9.0*	2.3	*8.6*	0.4	*0.4*
Macroplacoid row	4	4.0 – 6.5	*18.2* – *22.4*	5.5	*20.7*	1.1	*1.9*
Claw I heights							
External base	2	4.9 – 7.2	*22.1* – *24.4*	6.0	*23.3*	1.6	*1.7*
External primary branch	0	?	?	?	?	?	?
External secondary branch	2	4.6 – 7.4	*20.7* – *25.1*	6.0	*22.9*	2.0	*3.1*
Internal base	4	3.6 – 6.4	*16.2* – *21.8*	4.8	*18.1*	1.2	*2.6*
Internal primary branch	4	4.6 – 8.0	*20.4* – *27.3*	6.1	*22.7*	1.4	*3.1*
Internal secondary branch	4	4.0 – 5.4	*17.6* – *20.0*	4.9	*18.6*	0.6	*1.1*
Claw II heights							
External base	3	4.7 – 9.0	*21.2* – *30.5*	7.1	*26.4*	2.2	*4.8*
External primary branch	3	6.6 – 10.5	*30.0* – *36.1*	9.0	*33.9*	2.1	*3.4*
External secondary branch	3	4.6 – 7.0	*20.9* – *23.7*	5.9	*22.3*	1.2	*1.4*
Internal base	4	3.7 – 6.3	*15.4* – *21.3*	4.8	*18.0*	1.1	*2.6*
Internal primary branch	4	5.0 – 8.1	*21.4* – *27.4*	6.3	*23.8*	1.3	*2.6*
Internal secondary branch	4	4.1 – 6.6	*17.5* – *22.5*	5.2	*19.7*	1.1	*2.2*
Claw III heights							
External base	3	5.2 – 8.6	*23.3* – *29.3*	6.8	*25.7*	1.7	*3.2*
External primary branch	3	6.7 – 11.1	*30.6* – *37.6*	9.0	*34.1*	2.2	*3.5*
External secondary branch	3	4.2 – 6.6	*18.9* – *22.5*	5.5	*20.7*	1.2	*1.8*
Internal base	4	3.9 – 6.1	*16.8* – *22.4*	5.1	*19.1*	1.0	*2.5*
Internal primary branch	3	4.6 – 7.6	*20.8* – *25.9*	6.2	*23.6*	1.5	*2.6*
Internal secondary branch	4	3.6 – 7.0	*16.3* – *23.9*	5.1	*19.1*	1.4	*3.5*
Claw IV lengths							
Anterior base	1	4.5 – 4.5	*20.2* – *20.2*	4.5	*20.2*	?	?
Anterior primary branch	1	5.5 – 5.5	*24.9* – *24.9*	5.5	*24.9*	?	?
Anterior secondary branch	1	3.8 – 3.8	*17.4* – *17.4*	3.8	*17.4*	?	?
Posterior base	1	5.4 – 5.4	*24.4* – *24.4*	5.4	*24.4*	?	?
Posterior primary branch	0	?	?	?	?	?	?

Posterior secondary branch	1	4.2	–	4.2	*19.1*	–	*19.1*	4.2	*19.1*	?	?

Peribuccal lamellae and peribuccal papulae are absent. The oral cavity armature not visible in PCM. Buccal-pharyngeal apparatus of the *Ramazzottius* type with well visible thickening just below a stylet insertion point (Fig 49, filled unindented arrowhead). The pharyngeal bulb spherical with triangular apophyses and two macroplacoids (Figs 50–51). Microplacoid and septulum are absent. Macroplacoids roundish and without constrictions (Figs 50–51).

Figs 49–51. *Ramazzottius syraxi* sp. nov.: 49 – bucco-pharyngeal apparatus, dorsal view (holotype); 50 – placoid row, dorsal view (holotype); 51 – placoid row, ventral view (holotype). Filled unindented arrowhead represents thickening on buccal tube below a stylet insertion point. All PCM. Scale bars in μm.

Claws of the *Ramazzottius* type (Figs 52–53), i.e., two claws of the same leg clearly different from each other in size and shape. Primary branches of external claws and posterior claws longer than the primary branches of internal claws and anterior claws. The bases of all claws have a smooth pseudolunules (Figs 52–53, filled unindented arrowhead). Primary branches of external/posterior claws with cuticular flexible portions, connected to the secondary branches. Accessory points present on all primary branches (Figs 52–53, filled arrow).

Eggs unknown.

Figs 52–53. *Ramazzottius syraxi* sp. nov.: 52 – claws II (paratype); 53 – claws IV (paratype). Filled unindented arrowhead represents a smooth pseudolunule and filled arrow represents accessory points on primary branches of claws and. All PCM. Scale bars in μm.

Remarks

There have been efforts to obtain DNA sequences for *Ram. syraxi* sp. nov. Due to the small number of specimens, only two individuals were used for DNA sequencing to analyse the markers 18S, 28S, COI and ITS-2. In both cases, the sequences were contaminated or failed to produce reliable results. Therefore, we decided not to publish poor quality genetic data and focus solely on the morphological description.

Morphological differential diagnosis

Ramazzottius syraxi sp. nov. is characterized by the presence of gibbosities and well visible sculpture on dorsal side of the body and it is similar to *Ram. saltensis* (Claps and Rossi 1984) [77] and *Ram. szeptycki* (Dastych 1980) [78]. However, new species differs specifically from:

1. ***Ramazzottius saltensis***, known only from the type locality in Argentina [77] by: the different gibbosities configuration (VIII:1-2-3-4-5-4-3-1 in *Ram. syraxi vs* VIII:3-5-5-5-5-5-3-2 in *Ram. saltensis*).
2. ***Ramazzottius szeptycki***, known only from Republic of South Africa (type locality) and Tanzania [78-82] by: the different gibbosities configuration (VIII:1-2-3-4-5-4-3-1 in *Ram. syraxi vs* VII:8-8-10-10-10-10-4 in *Ram. szeptycki*) and different dorsal sculpture (smaller and larger polygonal granules in *Ram. syraxi vs* polygonal reticulation in *Ram. szeptycki*)

Conclusions

In mainland Ecuador, 27 species of tardigrades have been reported so far [31,38-41]. Of this number, only three species of the genus *Macrobiotus* were reported i.e. *Mac. hibiscus* De Barros 1942 [68], *Mac. polypiformis* and *Mac. dulciporus* Roszkowska, Gawlak, Draga & Kaczmarek 2019 [41]. *Macrobiotus hibiscus* was described from the type locality in Brazil

[68] and later reported from Ecuador [83], Tanzania [84], New Zeland [85], Argentina [77,86,87], China [88,89], and USA (see review in Kaczmarek et al. [32]; [90]). But due to the extremely limited original description, the species was classified as *nomina inquirenda* by Stec et al. [18]. That is why all current records of *Mac. hibiscus* should be considered as doubtful, and the species require detailed examination of the type material or new specimens from the type locality. In turn, *Mac. polypiformis* and *Mac. dulciporus* were described from Ecuador recently and are known only from their type localities [39,41].

Regarding genus *Mesobiotus*, only two species have been recorded in Ecuador i.e. *Meb. coronatus* and *Meb. romani* Roszkowska, Stec, Gawlak & Kaczmarek 2018 [40]. *Mesobiotus coronatus* was described from the type locality in Brazil [68] and later redescribed based on material from Ecuador [70]. This species has a relatively wide distribution: Costa Rica, Dominican Republic (Greater Antilles), Mexico (see review in Kaczmarek et al. [30]), Argentina, Chile, Peru, Uruguay (see review in Kaczmarek et al. [31]), USA (see review in Kaczmarek et al. [32]), New Zealand [85,91], Antarctica [92], Russia [93], China [94], Seychelles [72], Saint Martin (Lesser Antilles) [95]. Despite this, it was suggested by Pilato et al. [70] that true *Meb. coronatus* has confirmed localities only in South America. All other localities require a detailed reconsideration also since this species belongs to the *Mac. harmsworthi* group, which needs a really careful examination of specimens and eggs [54,66]. *Mesobiotus romani* was described from Ecuador recently and is known only from the type locality [40].

Ramazzottius syraxi sp. nov. is the first species of the genus *Ramazzottius* found in Ecuador.

Therefore, the number of known species from mainland Ecuador has increased to 30 with three new described species and much more species is waiting for discovery in this region.

Acknowledgments

We are grateful to Daniel Stec form Institute of Systematics and Evolution of Animals, Polish Academy of Sciences (Kraków, Poland) for kindly providing us help with analyzing of the DNA sequences.

Studies have been partially conducted in the framework of activities of BARg (Biodiversity and Astrobiology Research group).

References

1. Ministerio del Ambiente y Agua. Plan de Manejo del Parque Nacional Cotacachi Cayapas. 2020. Available from: https://www.conservation.org/ecuador/noticias/2020/11/17/el-parque-nacional-cotacachi-cayapas-ya-cuenta-con-su-nuevo-plan-de-manejo

2. Myers N, Mittermeier RA, Mittermeier CG, da Fonseca GAB, Kent J. Biodiversity hotspots for conservation priorities. Nature. 2000 Feb;403(6772): 853–858. doi: 10.1038/35002501

3. Cuenca P, Echeverria C. How do protected landscapes associated with high biodiversity and population levels change? PLOS ONE. 2017 Jul 3;12(7): e0180537. doi: 10.1371/journal.pone.0180537

4. Degma P, Guidetti R. Actual checklist of Tardigrada species (43rd Edition: 01-07-2024). 2009–2024. Available from: https://iris.unimore.it/handle/11380/1178608

5. Wełnicz W, Grohme MA, Kaczmarek Ł, Schill RO, Frohme M. Anhydrobiosis in tardigrades — The last decade. J Insect Physiol. 2011 May 1;57(5): 577–583. doi: 10.1016/j.jinsphys.2011.03.019

6. Guidetti R, Rizzo AM, Altiero T, Rebecchi L. What can we learn from the toughest animals of the Earth? Water bears (tardigrades) as multicellular model organisms in order to perform scientific preparations for lunar exploration. Planet Space Sci. 2012 Dec;74(1): 97–102. doi: 10.1016/j.pss.2012.05.021

7. Nelson DR, Guidetti R, Rebecchi L. Phylum Tardigrada. In: Throp JH, Rogers DC, editors. Thorp and Covich's Freshwater Invertebrates. Vol.1. Ecology and General Biology. Cambridge: Academic Press; 2015. p. 347–380. doi: 10.1016/B978-0-12-385026-3.00017-6

8. Thulin G. Über die Phylogenie und das system der Tardigraden. Hereditas. 1928;11(2–3): 207–266. doi: 10.1111/j.1601-5223.1928.tb02488.x

9. Schultze CAS. *Macrobiotus hufelandii* animal e crustaceorum classe novum, reviviscendi post diuturnam asphixiam et aridiatem potens. Berolini, Apud Carolum Curths. 1834: 1–7.

10. Vecchi M, Cesari M, Bertolani R, Jönsson KI, Rebecchi L, Guidetti R. Integrative systematic studies on tardigrades from Antarctica identify new genera and new species within Macrobiotoidea and Echiniscoidea. Invertebr Syst. 2016;30(4): 303–322. doi: 10.1071/IS15033

11. Schuster RO, Nelson DR, Grigarick AA, Christenberry D. Systematic Criteria of the Eutardigrada. Trans Am Micros Soc. 1980;99(3): 284–303. doi: 10.2307/3226004

12. Guidetti R, Schill RO, Bertolani R, Dandekar T, Wolf M. New molecular data for tardigrade phylogeny, with the erection of *Paramacrobiotus* gen. nov. J Zool Syst Evol Res. 2009 Nov;47(4): 315–321. doi: 10.1111/j.1439-0469.2009.00526.x

13. Guidetti R, Peluffo JR, Rocha AM, Cesari M, de Peluffo MCM. The morphological and molecular analyses of a new South American urban tardigrade offer new insights on the biological meaning of the *Macrobiotus hufelandi* group of species (Tardigrada: Macrobiotidae). J Nat Hist. 2013 Oct 1;47(37–38): 2409–2426. doi: 10.1080/00222933.2013.800610

14. Kaczmarek Ł, Michalczyk Ł. The *Macrobiotus hufelandi* group (Tardigrada) revisited. Zootaxa. 2017 Dec 8;4363(1): 101–123. doi: 10.11646/zootaxa.4363.1.4

15. Stec D, Vecchi M, Calhim S, Michalczyk Ł. New multilocus phylogeny reorganises the family Macrobiotidae (Eutardigrada) and unveils complex morphological evolution of the *Macrobiotus hufelandi* group. Mol Phylogenet Evol. 2021 Jul;160: 106987. doi: 10.1016/j.ympev.2020.106987

16. Kaczmarek Ł, Rutkowski T, Zacharyasiewicz M, Surmacki A, Osiejuk TS, Kayastha P. New species of Macrobiotidae (Eutardigrada) from Cameroon (Africa), characteristics of *Macrobiotus* morpho-groups and a key to the *nelsonae* group. Ann Zool. 2023 Mar;73(1): 1–15. doi: 10.3161/00034541ANZ2023.73.1.001

17. Massa E, Guidetti R, Cesari M, Rebecchi L, Jönsson KI. Tardigrades of Kristianstads Vattenrike Biosphere Reserve with description of four new species from Sweden. Sci Rep. 2021 Mar 1;11(1): 4861. doi: 10.1038/s41598-021-83627-w

18. Stec D, Vecchi M, Dudziak M, Bartels PJ, Calhim S, Michalczyk Ł. Integrative taxonomy resolves species identities within the *Macrobiotus pallarii* complex (Eutardigrada: Macrobiotidae). Zool Lett. 2021 May 27;7(1): 9. doi: 10.1186/s40851-021-00176-w

19. Stec D, Vončina K, Kristensen MR, Michalczyk Ł. The *Macrobiotus ariekammensis* species complex provides evidence for parallel evolution of claw elongation in macrobiotid tardigrades. Zool J Linn Soc. 2022 Jul 29;195(4): 1067–1099. doi: 10.1093/zoolinnean/zlab101

20. Vecchi M, Stec D. Integrative descriptions of two new *Macrobiotus* species (Tardigrada, Eutardigrada, Macrobiotidae) from Mississippi (USA) and Crete (Greece). Zoosyst Evol. 2021 May 19;97(1): 281–306. doi: 10.3897/zse.97.65280

21. Bertolani R, Cesari M, Giovannini I, Rebecchi L, Guidetti R, Kaczmarek Ł, Pilato G. The *Macrobiotus persimilis-polonicus* complex (Eutardigrada, Macrobiotidae), another example of problematic species identification, with the description of four new species. Org Divers Evol. 2023 Jun 1;23(2): 329–368. doi: 10.1007/s13127-022-00599-z

22. Tumanov DV, Androsova ED, Gavrilenko MD, Kalimullin AA. Integrative description of two new species of the genus *Mesobiotus* (Eutardigrada, Macrobiotoidea) from Russia, with an updated phylogeny of the genus. Eur J Taxon. 2024 Jul 30;947: 20–52. doi: 10.5852/ejt.2024.947.2619

23. Short KA, Sands CJ, McInnes SJ, Pisani D, Stevens MI, Convey P. An ancient, Antarctic-specific species complex: large divergences between multiple Antarctic lineages of the tardigrade genus *Mesobiotus*. Mol Phylogenet Evol. 2022 May 1;170: 107429. doi: 10.1016/j.ympev.2022.107429

24. Stec D. An integrative description of two new *Mesobiotus* species (Tardigrada: Eutardigrada: Macrobiotidae) with updated genus phylogeny. Zool Stud. 2022 Dec 15;61: 1–30. doi: 10.6620/ZS.2022.61-85

25. Sands CJ, McInnes SJ, Marley NJ, Goodall-Copestake WP, Convey P, Linse K. Phylum Tardigrada: an “individual” approach. Cladistics. 2008;24(6): 861–871. doi: 10.1111/j.1096-0031.2008.00219.x

26. Binda MG, Pilato G. *Ramazzottius*, nuovo genere di eutardigrado (Hypsibiidae). Animalia. 1986;13(1–3): 159–166.

27. Doyère L. Mémoire sur les Tardigrades. Ann Sci Nat. 1840;14: 269–361.

28. Pilato G, Binda MG. Definition of families, subfamilies, genera and subgenera of the Eutardigrada, and keys to their identification. Zootaxa. 2010 Mar 22;2404(1): 1–54. doi: 10.11646/zootaxa.2404.1.1

29. McInnes SJ. Zoogeographic distribution of terrestrial/freshwater tardigrades from current literature. J Nat Hist. 1994 Apr 1;28(2): 257–352. doi: 10.1080/00222939400770131

30. Kaczmarek Ł, Michalczyk Ł, McInnes SJ. Annotated zoogeography of non-marine Tardigrada. Part I: Central America. Zootaxa. 2014 Feb 5;3763(1): 1–62. doi: 10.11646/zootaxa.3763.1.1

31. Kaczmarek Ł, Michalczyk Ł, McInnes SJ. Annotated zoogeography of non-marine Tardigrada. Part II: South America. Zootaxa. 2015 Feb 25;3923: 1–107. doi: 10.11646/zootaxa.3923.1.1

32. Kaczmarek Ł, Michalczyk Ł, Mcinnes SJ. Annotated zoogeography of non-marine Tardigrada. Part III: North America and Greenland. Zootaxa. 2016 Dec 1;4203(1): 1–249. doi: 10.11646/zootaxa.4203.1.1

33. McInnes SJ, Michalczyk Ł, Kaczmarek Ł. Annotated zoogeography of non-marine Tardigrada. Part IV: Africa. Zootaxa. 2017 Jun 28;4284(1): 1–74. doi: 10.11646/zootaxa.4284.1.1

34. Kihm J-H, Zawierucha K, Rho HS, Park T-YS. Homology of the head sensory structures between Heterotardigrada and Eutardigrada supported in a new species of water bear (Ramazzottiidae: *Ramazzottius*). Zool Lett. 2023 Nov 27;9(1): 22. doi: 10.1186/s40851-023-00221-w

35. Dey PK, López-López A, Morek W, Michalczyk Ł. Tardigrade Augean stables — a challenging phylogeny and taxonomy of the family Ramazzottiidae (Eutardigrada: Hypsibioidea). Zool J Linn Soc. 2024 Jan 1;200(1): 95–110. doi: 10.1093/zoolinnean/zlad161

36. Stec D, Morek W, Gąsiorek P, Michalczyk Ł. Unmasking hidden species diversity within the *Ramazzottius oberhaeuseri* complex, with an integrative redescription of the nominal species for the family Ramazzottiidae (Tardigrada: Eutardigrada: Parachela). Syst Biodivers. 2018 May 19;16(4): 357–376. doi: 10.1080/14772000.2018.1424267

37. Guidetti R, Cesari M, Giovannini I, Ebel C, Förschler MI, Rebecchi L, Schill RO. Morphology and taxonomy of the genus *Ramazzottius* (Eutardigrada; Ramazzottiidae) with the integrative description of *Ramazzottius kretschmanni* sp. nov. Eur Zool J. 2022 Dec 31;89(1): 346–370. doi: 10.1080/24750263.2022.2043468

38. Kaczmarek Ł, Gawlak M, Bartels PJ, Nelson DR, Roszkowska M. Revision of the genus *Paramacrobiotus* Guidetti et al., 2009 with the description of a new species, re-descriptions and a key. Ann Zool. 2017 Dec;67(4): 627–656. doi: 10.3161/00034541ANZ2017.67.4.001

39. Roszkowska M, Ostrowska M, Stec D, Janko K, Kaczmarek Ł. *Macrobiotus polypiformis* sp. nov., a new tardigrade (Macrobiotidae; *hufelandi* group) from the Ecuadorian Pacific coast, with remarks on the claw abnormalities in eutardigrades. Eur J Taxon. 2017 Jun 7;327: 1–19. doi: 10.5852/ejt.2017.327

40. Roszkowska M, Stec D, Gawlak M, Kaczmarek Ł. An integrative description of a new tardigrade species *Mesobiotus romani* sp. nov. (Macrobiotidae: *harmsworthi* group) from the Ecuadorian Pacific coast. Zootaxa. 2018 Jul 27;4450(5): 550–564. doi: 10.11646/zootaxa.4450.5.2

41. Roszkowska M, Gawlak M, Draga M, Kaczmarek Ł. Two new species of Tardigrada from Ecuador (South America). Zootaxa. 2019 Jan 21;4545(4): 511–530. doi: 10.11646/zootaxa.4545.4.4

42. Stec D, Smolak R, Kaczmarek Ł, Michalczyk Ł. An integrative description of *Macrobiotus paulinae* sp. nov. (Tardigrada: Eutardigrada: Macrobiotidae: *hufelandi* group) from Kenya. Zootaxa. 2015 Dec 7;4052(5): 501–536. doi: 10.11646/zootaxa.4052.5.1

43. Michalczyk Ł, Kaczmarek Ł. A description of the new tardigrade *Macrobiotus reinhardti* (Eutardigrada: Macrobiotidae, *harmsworthi* group) with some remarks on the oral cavity armature within the genus *Macrobiotus* Schultze. Zootaxa. 2003 Oct 21;331(1): 1–24. doi: 10.11646/zootaxa.331.1.1

44. Kaczmarek Ł, Cytan J, Zawierucha K, Diduszko D, Michalczyk Ł. Tardigrades from Peru (South America), with descriptions of three new species of Parachela. Zootaxa. 2014 Apr 17;3790(2): 357–379. doi: 10.11646/zootaxa.3790.2.5

45. Kiosya Y, Pogwizd J, Matsko Y, Vecchi M, Stec D. Phylogenetic position of two *Macrobiotus* species with a revisional note on *Macrobiotus sottilei* Pilato, Kiosya, Lisi & Sabella, 2012 (Tardigrada: Eutardigrada: Macrobiotidae). Zootaxa. 2021 Feb 18;4933(1): 113–135. doi: 10.11646/zootaxa.4933.1.5

46. Beasley CW, Kaczmarek Ł, Michalczyk Ł. *Doryphoribius mexicanus*, a new species of Tardigrada (Eutardigrada: Hypsibiidae) from Mexico (North America). Proc Biol Soc Wash. 2008 Apr;121(1): 34–40. doi: 10.2988/07-30.1

47. Pilato G. Analisi di nuovi caratteri nello studio degli Eutardigradi. Animalia. 1981;8: 51–57.

48. Michalczyk Ł, Kaczmarek Ł. The Tardigrada Register: a comprehensive online data repository for tardigrade taxonomy. J Limnol. 2013 May 3;72(s1): 175–181. doi: 10.4081/jlimnol.2013.s1.e22

49. Perry E, Miller WR, Kaczmarek Ł. Recommended abbreviations for the names of genera of the phylum Tardigrada. Zootaxa. 2019 May 17;4608(1): 145–154. doi: 10.11646/zootaxa.4608.1.8

50. Casquet J, Thebaud C, Gillespie RG. Chelex without boiling, a rapid and easy technique to obtain stable amplifiable DNA from small amounts of ethanol-stored spiders. Mol Ecol Resour. 2012;12(1): 136–141. doi: 10.1111/j.1755-0998.2011.03073.x

51. Folmer O, Black M, Hoeh W, Lutz R, Vrijenhoek R. DNA primers for amplification of mitochondrial cytochrome c oxidase subunit I from diverse metazoan invertebrates. Mol Mar Biol Biotechnol. 1994 Oct;3(5): 294–299.

52. Mironov SV, Dabert J, Dabert M. A new feather mite species of the genus *Proctophyllodes* Robin, 1877 (Astigmata: Proctophyllodidae) from the Long-tailed Tit *Aegithalos caudatus* (Passeriformes: Aegithalidae) — morphological description with DNA barcode data. Zootaxa. 2012 Apr 2;3253(1): 54–61. doi: 10.11646/zootaxa.3253.1.2

53. White TJ, Bruns T, Lee S, Taylor J. Amplification and direct sequencing of fungal ribosomal RNA genes for phylogenetics. In: Innis MA, Gelfand DH, Sninsky JJ, White TJ, editors. PCR Protocols: A Guide to Methods and Applications. Cambridge: Academic Press; 1990. p. 315–322. doi: 10.1016/B978-0-12-372180-8.50042-1

54. Kaczmarek Ł, Bartylak T, Stec D, Kulpa A, Kepel M, Kepel A, Roszkowska M. Revisiting the genus *Mesobiotus* Vecchi et al., 2016 (Eutardigrada, Macrobiotidae) — remarks, updated dichotomous key and an integrative description of new species from Madagascar. Zool Anz. 2020 Jul;287: 121–146. doi: 10.1016/j.jcz.2020.05.003

55. Altschul SF, Gish W, Miller W, Myers EW, Lipman DJ. Basic local alignment search tool. J Mol Biol. 1990 Oct 5;215(3): 403–410. doi: 10.1016/S0022-2836(05)80360-2

56. Katoh K, Misawa K, Kuma K, Miyata T. MAFFT: a novel method for rapid multiple sequence alignment based on fast Fourier transform. Nucleic Acids Res. 2002 Jul 15;30(14): 3059–3066. doi: 10.1093/nar/gkf436

57. Katoh K, Toh H. Recent developments in the MAFFT multiple sequence alignment program. Brief Bioinform. 2008 Jul 1;9(4): 286–298. doi: 10.1093/bib/bbn013

58. Kumar S, Stecher G, Li M, Knyaz C, Tamura K. MEGA X: Molecular evolutionary genetics analysis across computing platforms. Mol Biol Evol. 2018 Jun 1;35(6): 1547–1549. doi: 10.1093/molbev/msy096

59. Richters F. Tardigrada. In: Kükenthal W, Krumbach T, editors. Handbuch der Zoologie. Berlin: De Gruyter; 1926. p. 1–68.

60. Stec D, Arakawa K, Michalczyk Ł. An integrative description of *Macrobiotus shonaicus* sp. nov. (Tardigrada: Macrobiotidae) from Japan with notes on its phylogenetic position within the *hufelandi* group. PLOS ONE. 2018 Feb 28;13(2): e0192210. doi: 10.1371/journal.pone.0192210

61. Vecchi M, Stec D, Vuori T, Ryndov S, Chartrain J, Calhim S. *Macrobiotus naginae* sp. nov., a New Xerophilous Tardigrade Species from Rokua Sand Dunes (Finland). Zool Stud. 2022 May 30;61: e22. doi: 10.6620/ZS.2022.61-22

62. Stec D, Kristensen RM, Michalczyk Ł. Integrative taxonomy identifies *Macrobiotus papei*, a new tardigrade species of the *Macrobiotus hufelandi* complex (Eutardigrada: Macrobiotidae) from the Udzungwa Mountains National Park (Tanzania). Zootaxa. 2018 Jul 17;4446(2): 273–291. doi: 10.11646/zootaxa.4446.2.7

63. Sugiura K, Arakawa K, Matsumoto M. Distribution of *Macrobiotus shonaicus* Stec, Arakawa & Michalczyk, 2018 (Tardigrada: Eutardigrada: Macrobiotidae) in Japan. Zootaxa. 2020 Apr 23;4767(1): 56–70. doi: 10.11646/zootaxa.4767.1.2

64. Itang LAM, Stec D, Mapalo MA, Mirano-Bascos D, Michalczyk Ł. An integrative description of *Mesobiotus dilimanensis,* a new tardigrade species from the Philippines (Eutardigrada: Macrobiotidae: *furciger* group). Raffles Bull Zool. 2020;68: 1931. doi: 10.26107/RBZ-2020-0003

65. Murray J. XXV. — Arctic Tardigrada, collected by Wm. S. Bruce. Trans. R. Soc. Edinb. 1907;45(25): 669–681. doi: 10.1017/S0080456800011789

66. Kaczmarek Ł, Zawierucha K, Buda J, Stec D, Gawlak M, Michalczyk Ł, Roszkowska M. An integrative redescription of the nominal taxon for the *Mesobiotus harmsworthi* group (Tardigrada: Macrobiotidae) leads to descriptions of two new *Mesobiotus* species from Arctic. PLOS ONE. 2018 Oct 17;13(10): e0204756. doi: 10.1371/journal.pone.0204756

67. Stec D. Integrative descriptions of two new *Mesobiotus* species (Tardigrada, Eutardigrada, Macrobiotidae) from Vietnam. Diversity. 2021 Nov 21;13(11): 605. doi: 10.3390/d13110605

68. De Barros R. Tardígrados do estado de São Paulo, Brasil. II. Gênero 'Macrobiotus'. Rev Bras Biol. 1942;2(4): 373–386.

69. Pilato G, Sabella G, Lisi O. Two new tardigrade species from Sicily. Zootaxa. 2014 Jan 14;3754(2): 173–184. doi: 10.11646/zootaxa.3754.2.6

70. Pilato G, Binda MG, Napolitano A, Moncada E. The specific value of *Macrobiotus coronatus* De Barros 1942, and description of two new species of the *harmsworthi* group (Eutardigrada). Boll Delle Sedute Della Accad Gioenia Sci Nat Catania. 2000;33: 103–120.

71. Mapalo MA, Stec D, Mirano-Bascos D, Michalczyk Ł. *Mesobiotus philippinicus* sp. nov., the first limnoterrestrial tardigrade from the Philippines. Zootaxa. 2016 Jun 20;4126(3): 411–426. doi: 10.11646/zootaxa.4126.3.6

72. Pilato G, Binda MG, Lisi O. Three new species of eutardigrades from the Seychelles. N Z J Zool. 2006 Jan 1;33(1): 39–48. doi: 10.1080/03014223.2006.9518429

73. Michalczyk Ł, Kaczmarek Ł, Mcinnes SJ. Annotated zoogeography of non-marine Tardigrada. Part V: Australasia. Zootaxa. 2022 Mar 8;5107(1): 1–119. doi: 10.11646/zootaxa.5107.1.1

74. Schuster R, Greven H. A long-term study of population dynamics of tardigrades in the moss *Rhytidiadelphus squarrosus* (Hedw.) Warnst. J Limnol. 2007 Sep 1;66(s1): 141–151. doi: 10.4081/jlimnol.2007.s1.141

75. Pilato G. Evoluzione e nuova sistemazione degli eutardigrada. Boll Zool. 1969 Jan 1;36(3): 327–345. doi: 10.1080/11250006909436925

76. Marley NJ, Mcinnes SJ, Sands CJ. Phylum Tardigrada: A re-evaluation of the Parachela. Zootaxa. 2011 Apr 14;2819(1): 51–64. doi: 10.11646/zootaxa.2819.1.2

77. Claps MC, Rossi GC. Contribucion al conocimiento de los tardigrados de Argentina. IV. Acta Zool Lilloana. 1984;38: 45–50.

78. Dastych H. *Hypsibius szeptycki* sp. nov., a new species of Tardigrada from South Africa. Bull Académie Pol Sci. 1980;27: 505–508.

79. Dastych H. Notes on the African limno-terrestrial tardigrade *Ramazzottius szeptycki* (Dastych, 1980) (Tardigrada). Entomol Mitteilungen Aus Dem Zool Mus Hambg. 2009;15: 87–91.

80. Binda MG, Pilato G. Some notes on African tardigrades with a description of two new species. Trop Zool. 1995 Nov;8(2): 367–372. doi: 10.1080/03946975.1995.10539294

81. Pilato G, Binda MG, Catanzaro R. Remarks on some tardigrades of the African fauna with the description of three new species of Macrobiotus Schultze 1834. Trop Zool. 1991 Dec 1;4(2): 167–178. doi: 10.1080/03946975.1991.10539487

82. Van Rompu EA, De Smet WH. Some freshwater tardigrades from the Kilimanjaro. Natuurwet Tijdschr. 1991 Jan 1;73: 55–62.

83. Schuster RO, Grigarick AA. Tardigrada from the Galápagos and Cocos Islands. Proc Calif Acad Sci. 1966;34: 315–328.

84. Grigarick AA, Schuster RO, Toftner EC. Descriptive morphology of eggs of some species in the *Macrobiotus hufelandi* group (Tardigrada: Macrobiotidae). Pan-Pac Entomol. 1973;49(3): 258–263.

85. Horning DS, Schuster RO, Grigarick AA. Tardigrada of New Zealand. N Z J Zool. 1978 Jun 1;5(2): 185–280. doi: 10.1080/03014223.1978.10428316

86. Rossi GC, Claps MC. Contribucion al conocimiento de los tardigrados de Argentina. I. Rev Soc Entomol Arg. 1980;39: 243–250.

87. Claps MC, Rossi GC. Contribucion al conocimiento de los tardigrados de Argentina. II. Rev Soc Entomol Arg. 1981;40(1–4): 107–114.

88. Yang T. Tardigrades from some mosses of Shennong frame state forest parking in China. Acta Zootaxon Sin. 2007;32(1): 186–189.

89. Wang L. Tardigrades from the Yunnan-Guizhou Plateau (China) with description of two new species in the genera *Mixibius* (Eutardigrada: Hypsibiidae) and *Pseudechiniscus* (Heterotardigrada: Echiniscidae). J Nat Hist. 2009 Oct 5;43(41–42): 2553–2570. doi: 10.1080/00222930903221547

90. Cortes LET, Cortes BW, Miller WR. Tardigrades of Hardin County, Iowa: seven new records from Iowa, USA. Trans Kans Acad Sci. 2020 May;123(1–2): 203–212. doi: 10.1660/062.123.0117

91. Nelson DR, Horning DS. Tardigrada of the Kowhai Bush, Kaikoura, New Zealand. Zesz Nauk Uniw Jagiell. 1979;25: 125–142.

92. Utsugi K, Ohyama Y. Antarctica Tardigrada II. Molodezhnaya and Mt. Riiser-Larsen Areas. Proc NIPR Symp Polar Biol. 4: 161–170.

93. Biserov V. An annotated list of Tardigrada from European Russia. Zool Jahrb Abt Für Syst Geogr Biol Tiere. 1991;118: 193–216.

94. Yang T. Two new species and three new records of Tardigrada (Heterotardigrada, Echiniscidae; Eutardigrada, Milnesiidae, Macrobiotidae, Hypsibiidae). Acta Zootaxon Sin. 2003;28(2): 235–240.

95. Meyer HA. First record of water bears (Phylum Tardigrada) from Saint Martin, Lesser Antilles, West Indies. Tex J Sci. 2014;63(4): 309–312.

Supporting information

S1 Table. Raw morphometric data for *Macrobiotus sharopovi* sp. nov.

S2 Table. Raw morphometric data for *Mesobiotus shevchenkoi* sp. nov.

S3 Table. Raw morphometric data for *Ramazzottius syraxi* sp. nov. from the type locality.

S4 Table. Raw morphometric data for *Ramazzottius syraxi* sp. nov. from the additional locality.

S5 Table. Genetic distances for *Macrobiotus sharopovi* sp. nov.

S6 Table. Genetic distances for *Mesobiotus shevchenkoi* sp. nov.

Figs1-4 Click here to access/download;Figure;Figs1-4.tif

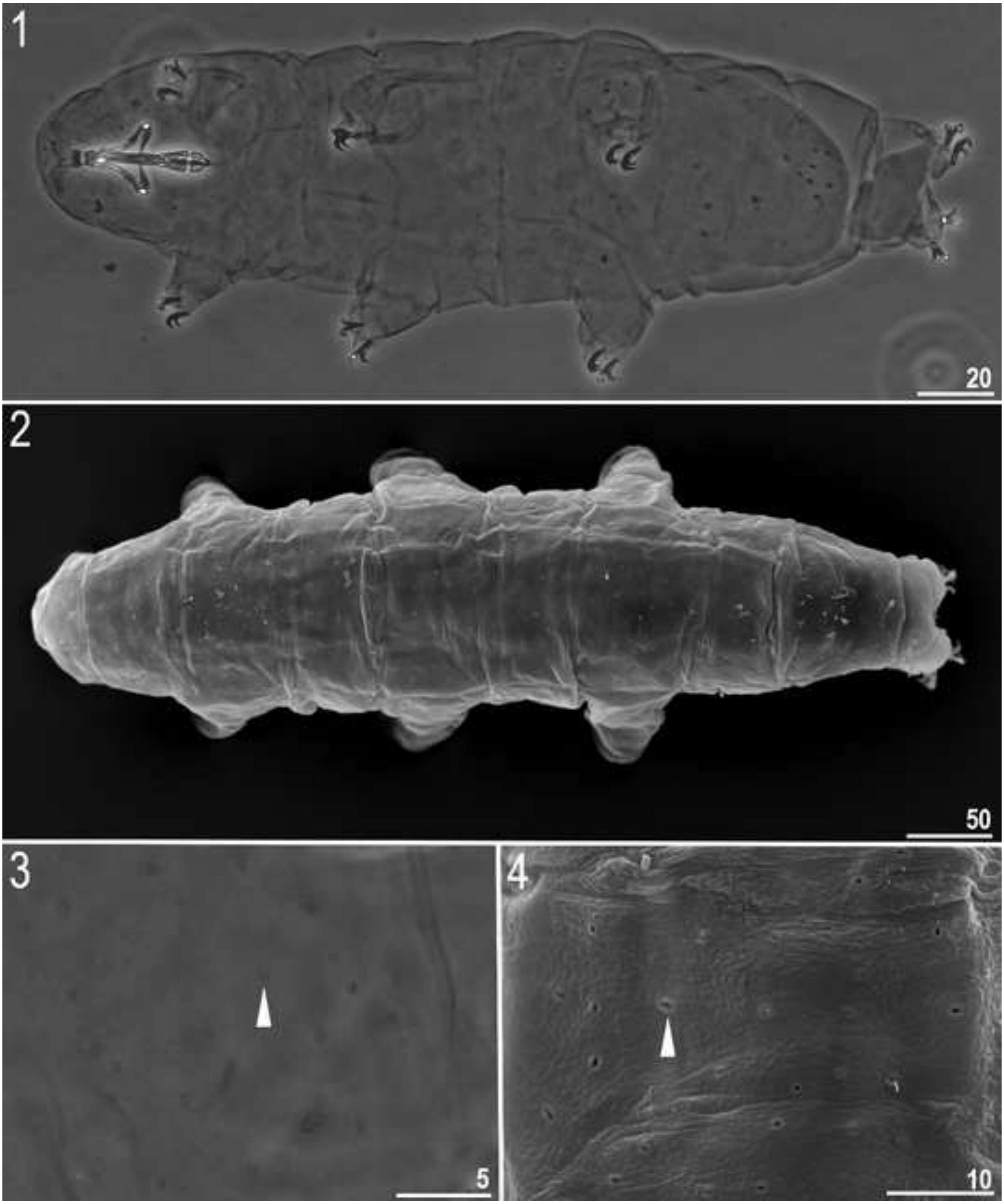

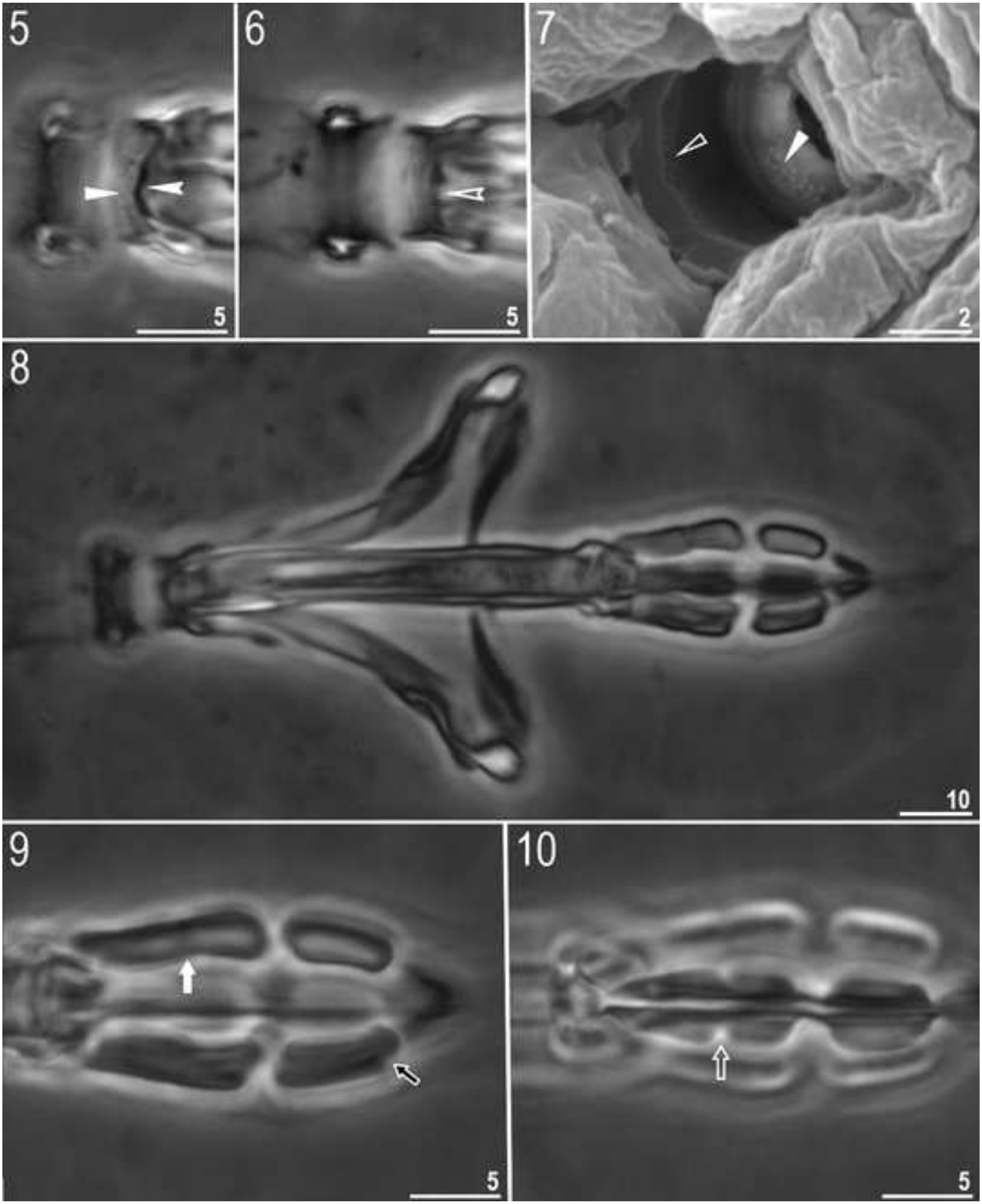
5
6
7
8
9
10
5
5
2
10
5
5

Figs11-14

Click here to access/download;Figure;Figs11-14.tif

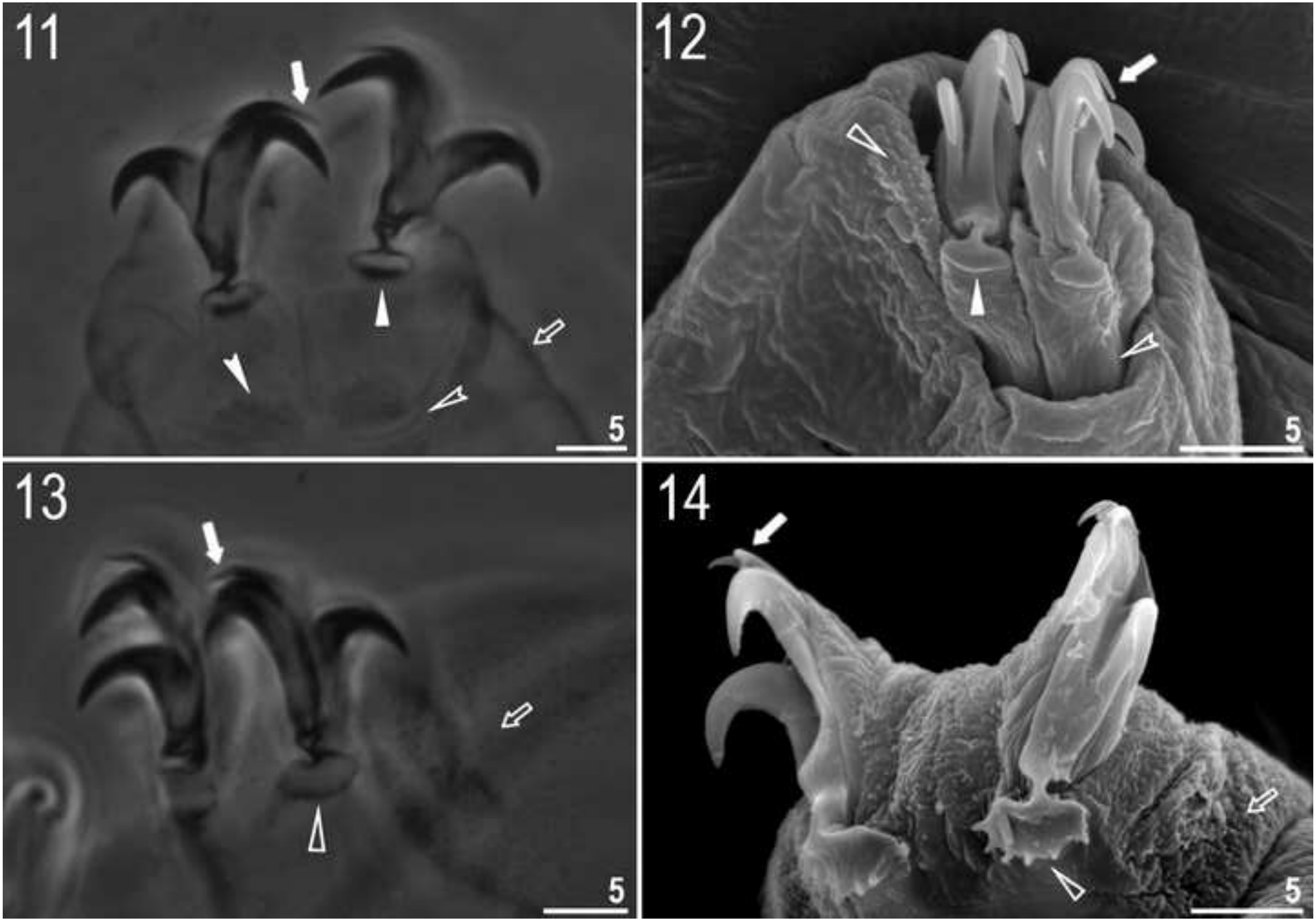

Figs15-21 Click here to access/download;Figure;Figs15-21.tif

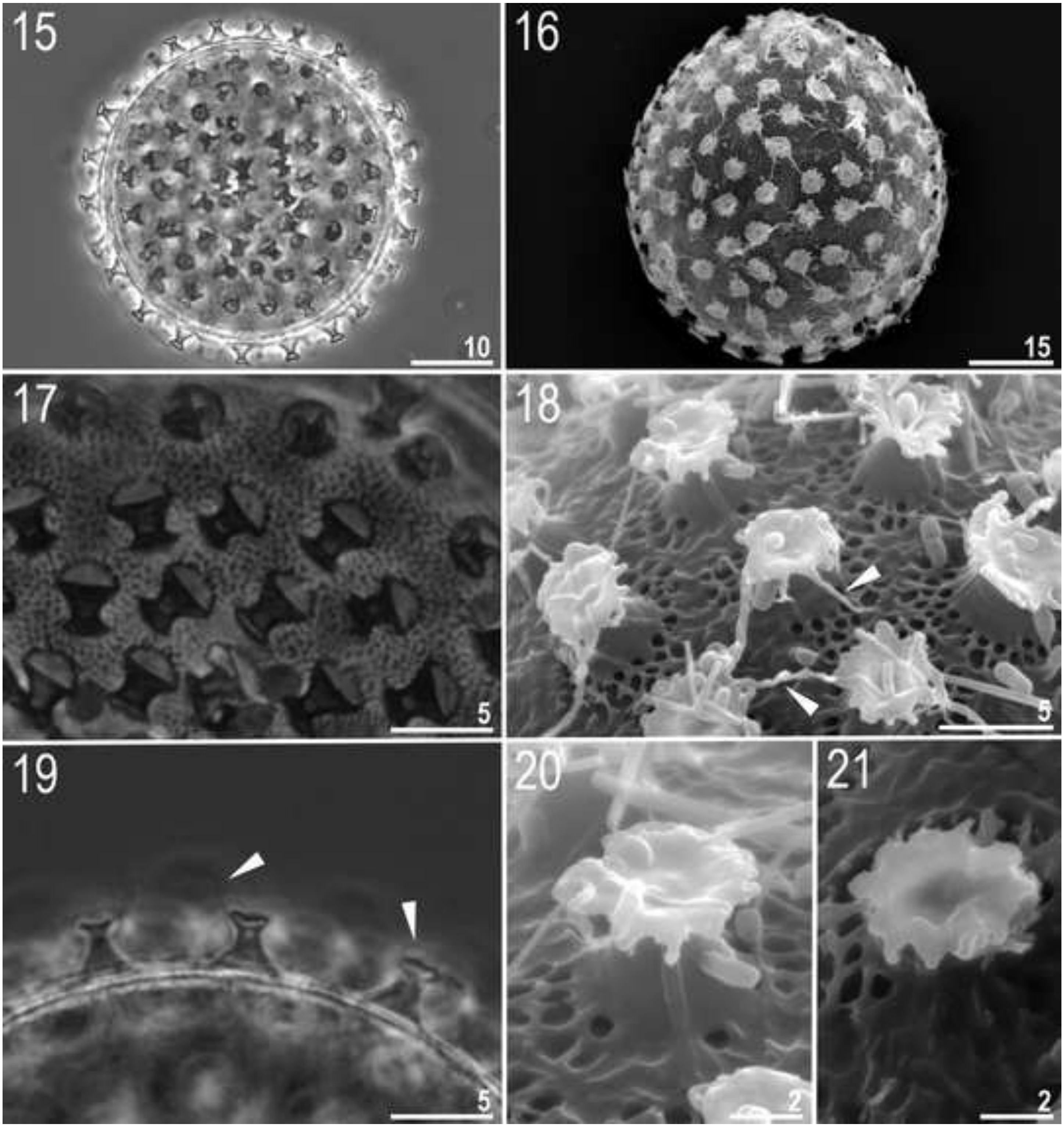

Figs22-26 Click here to access/download;Figure;Figs22-26.tif

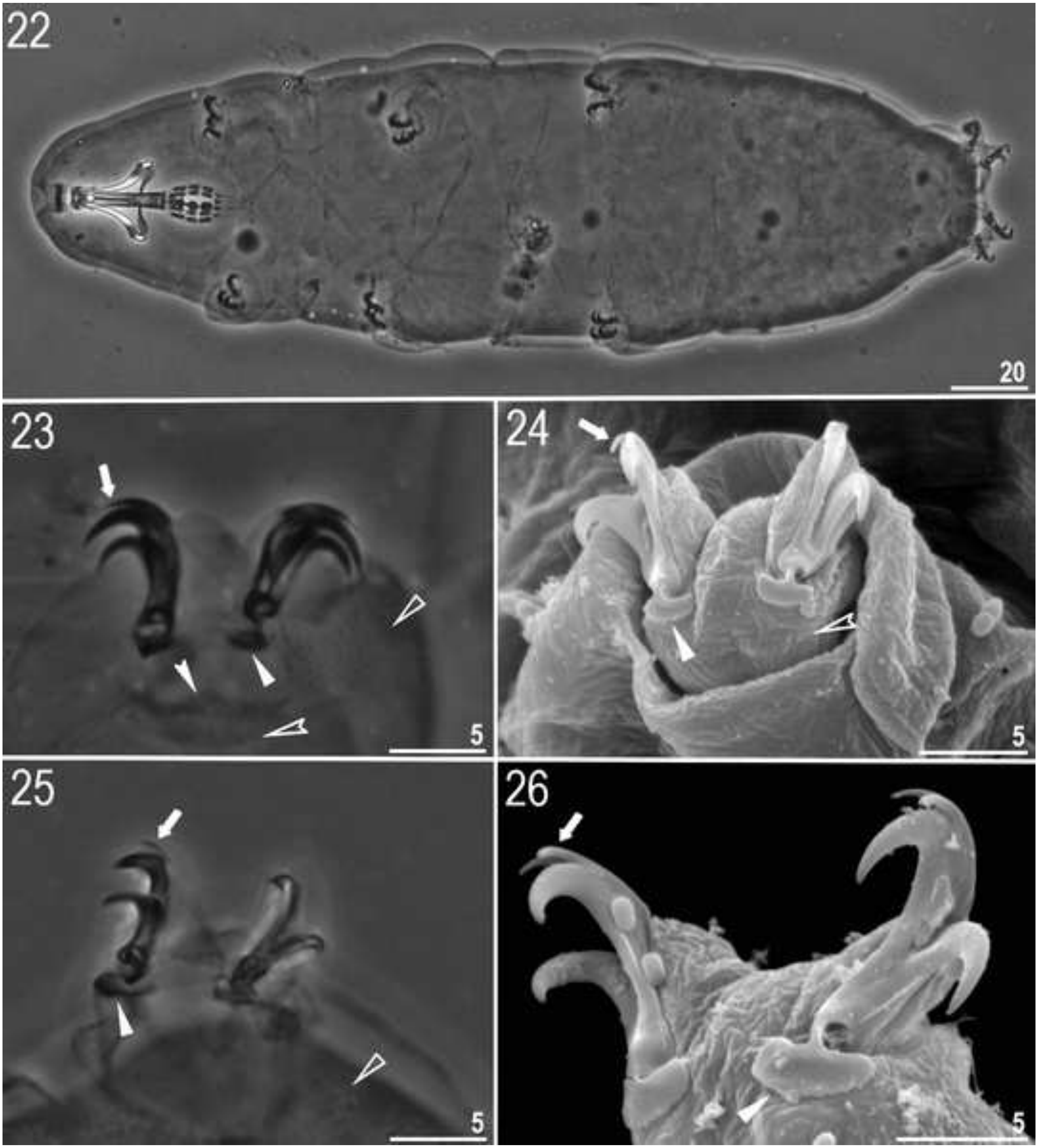

Figs27-31

Click here to access/download;Figure;Figs27-31.tif

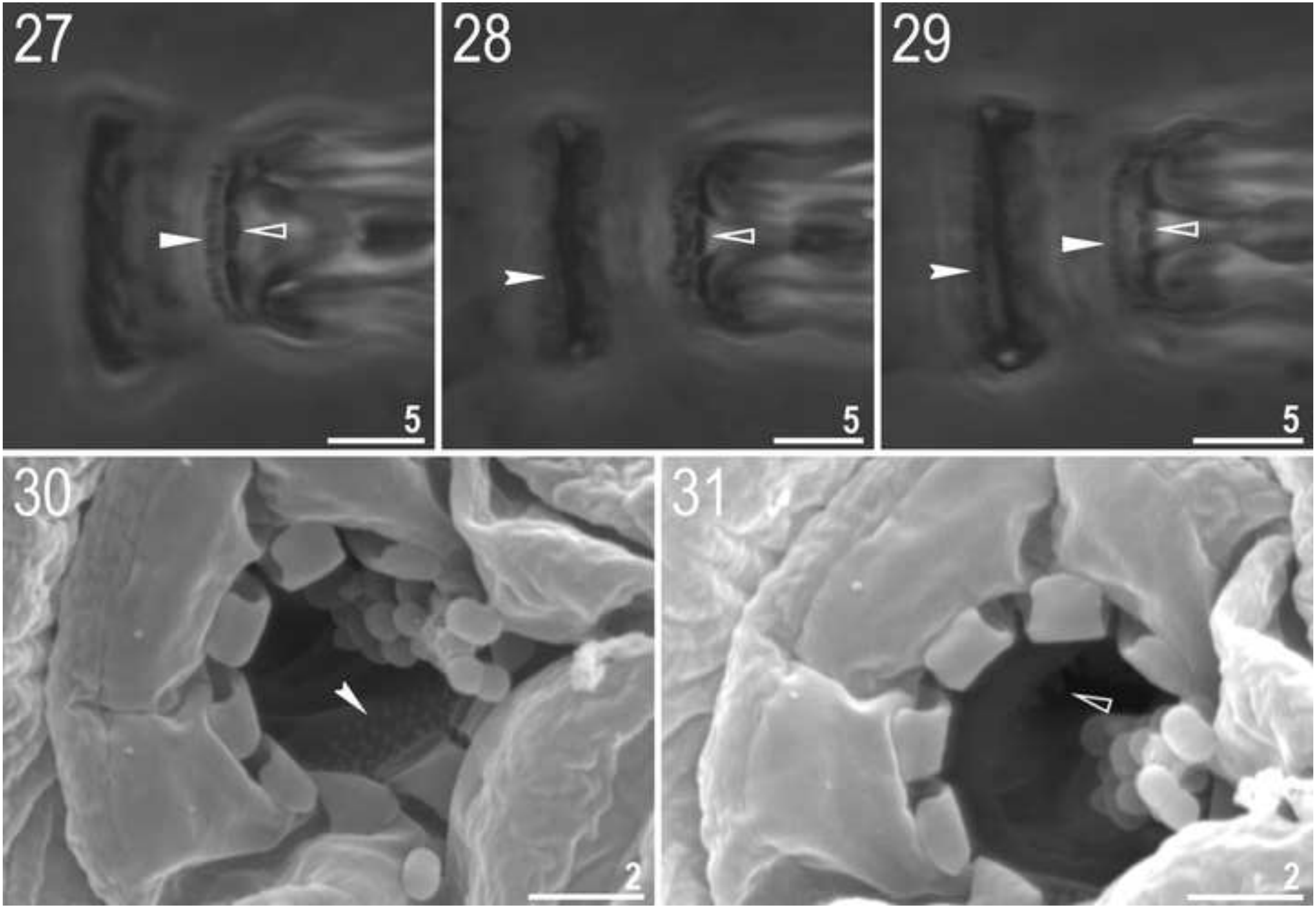

 Click here to access/download;Figure;Figs32-34.tif

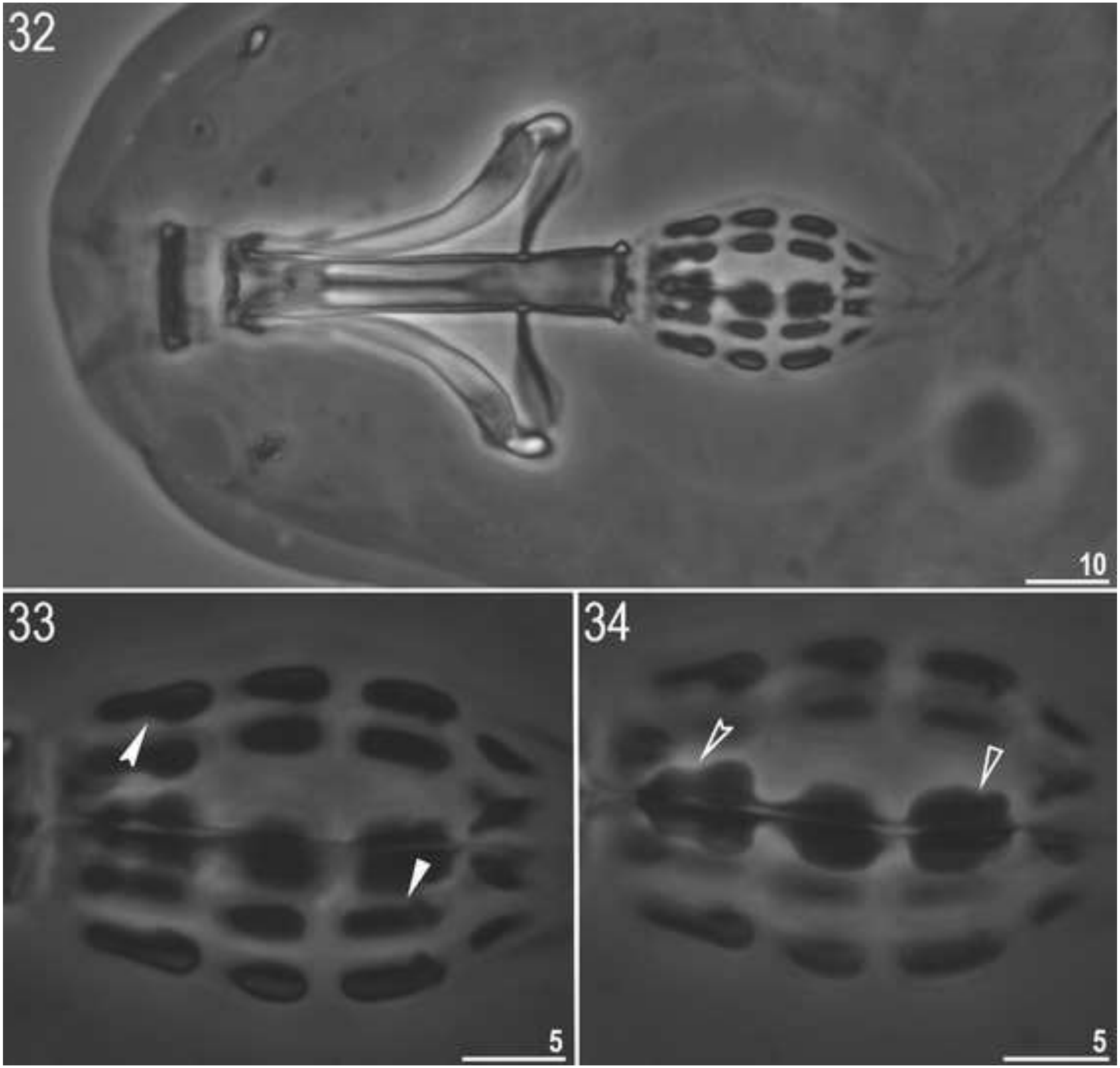

 Click here to access/download;Figure;Figs35-41.tif

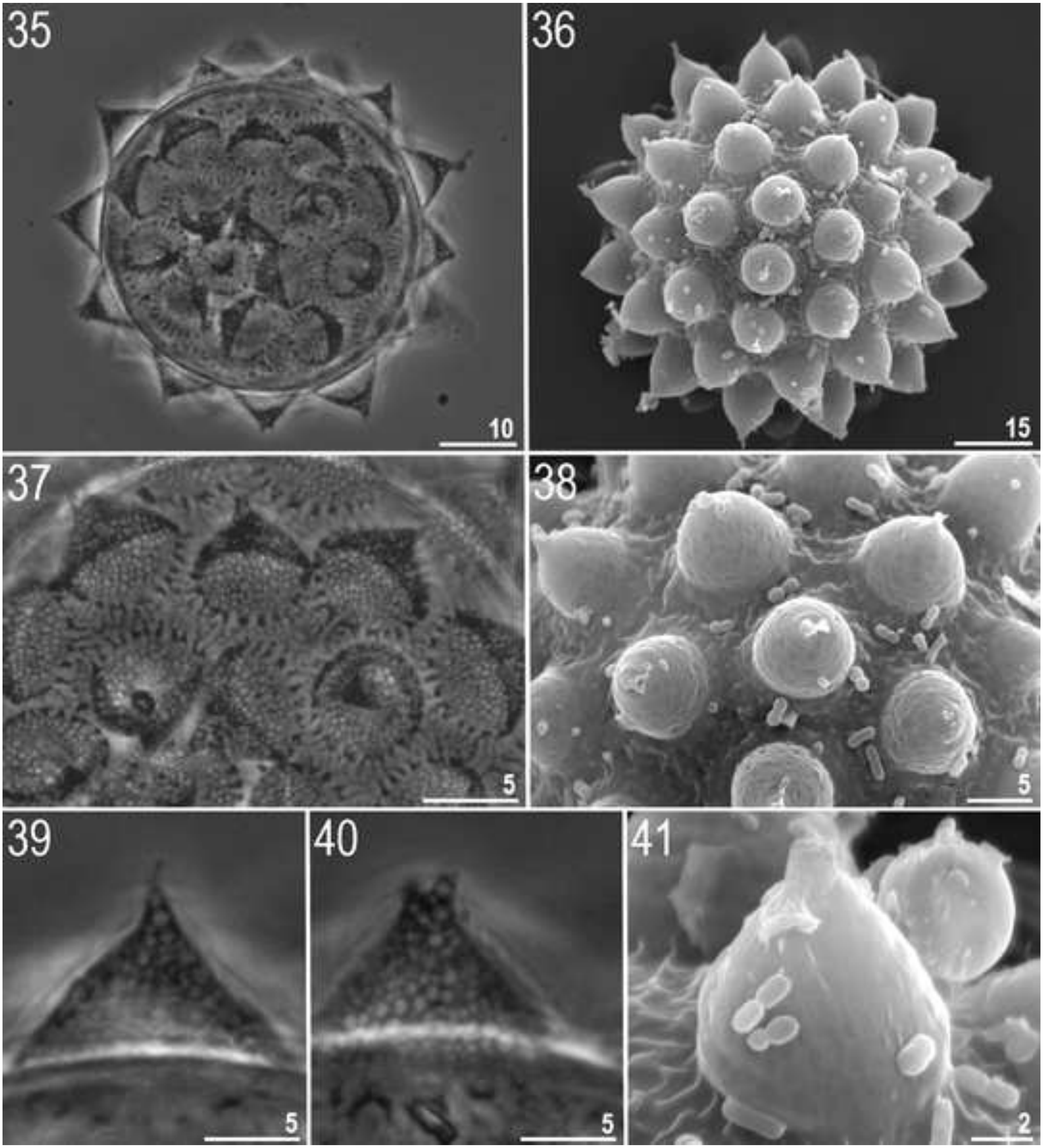

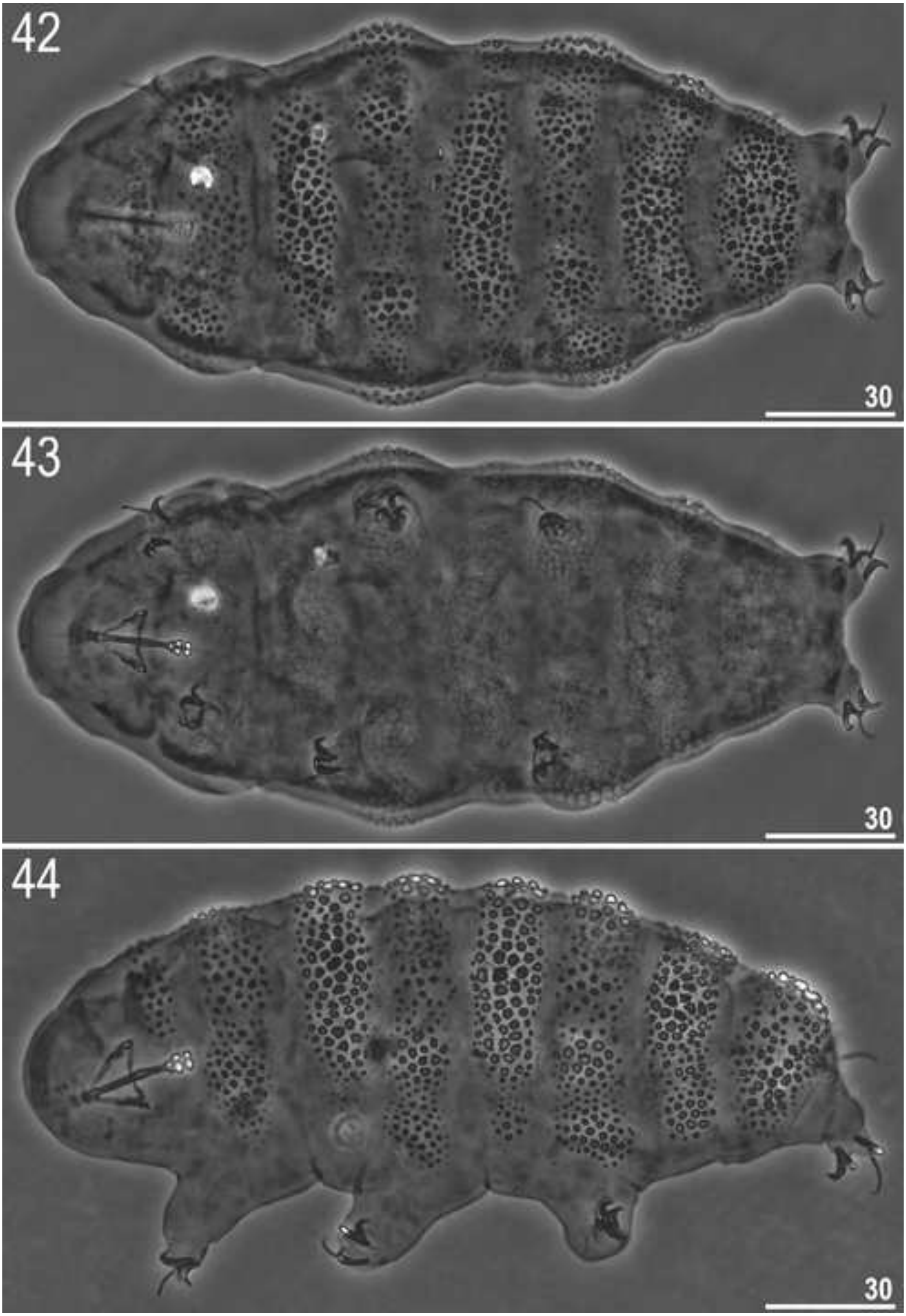
42
30
43
30
44
30

Click here to access/download;Figure;Figs45-48.tif

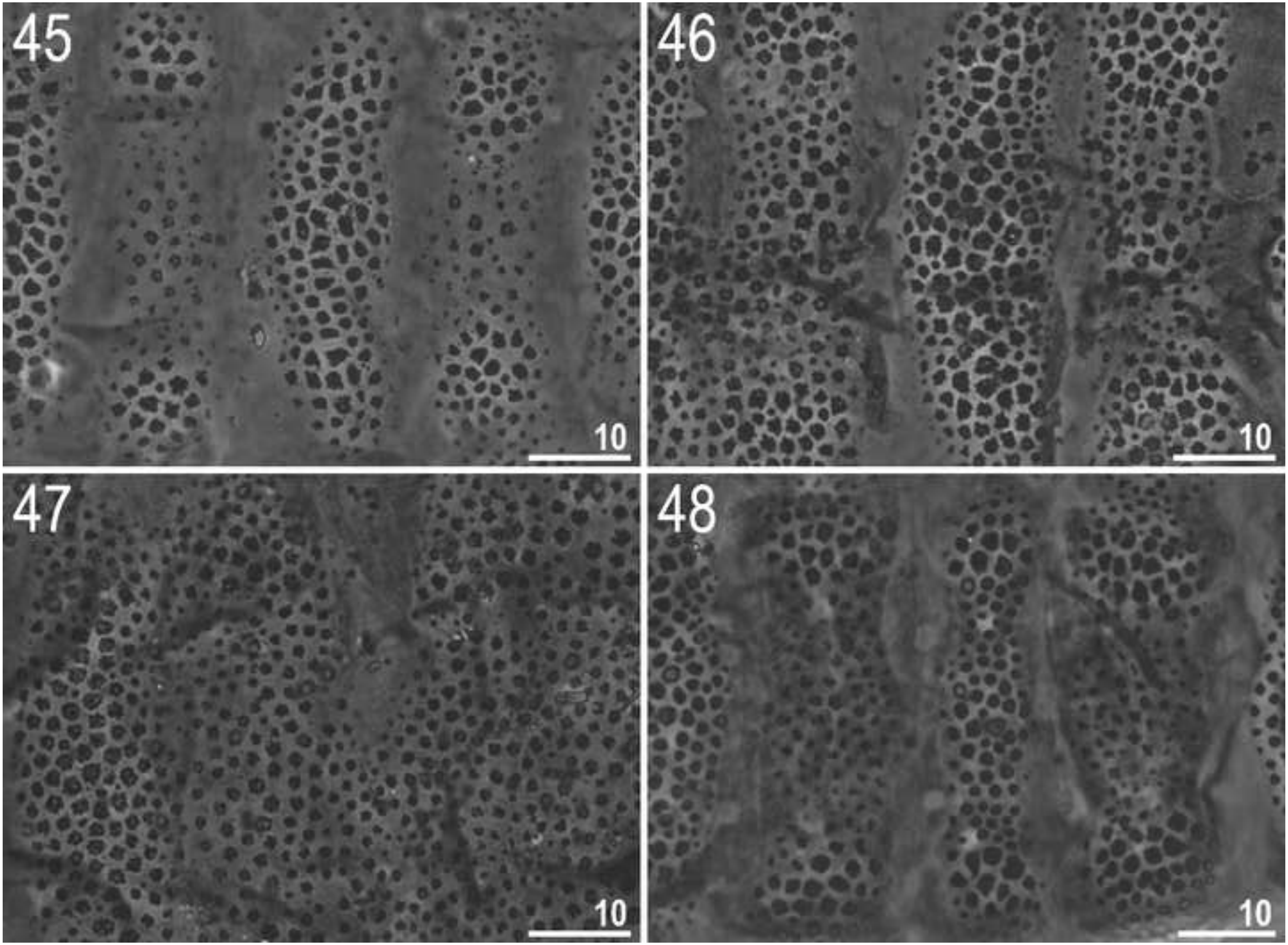

Click here to access/download;Figure;Figs49-51.tif

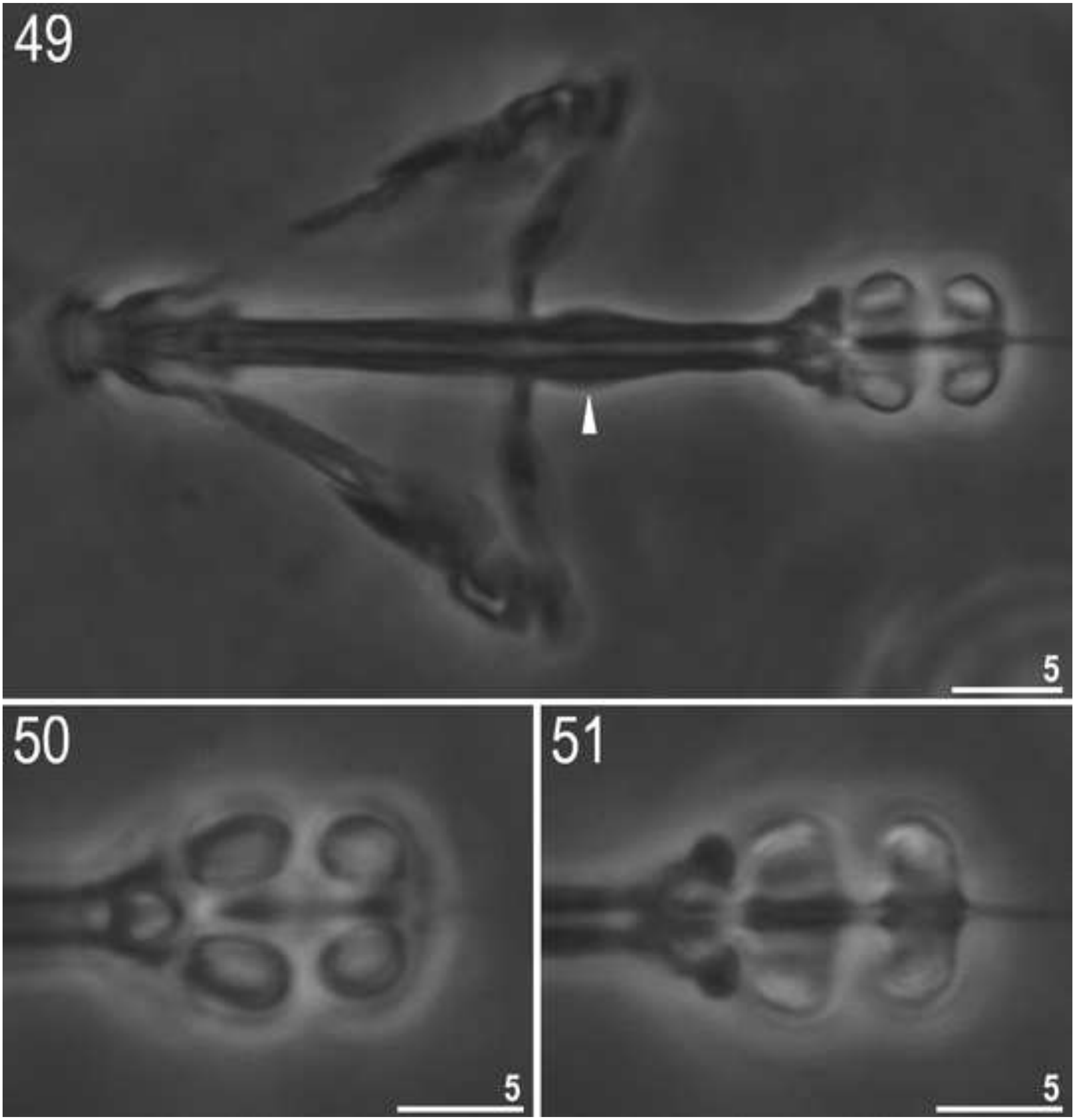

Figs52-53

Click here to access/download;Figure;Figs52-53.tif

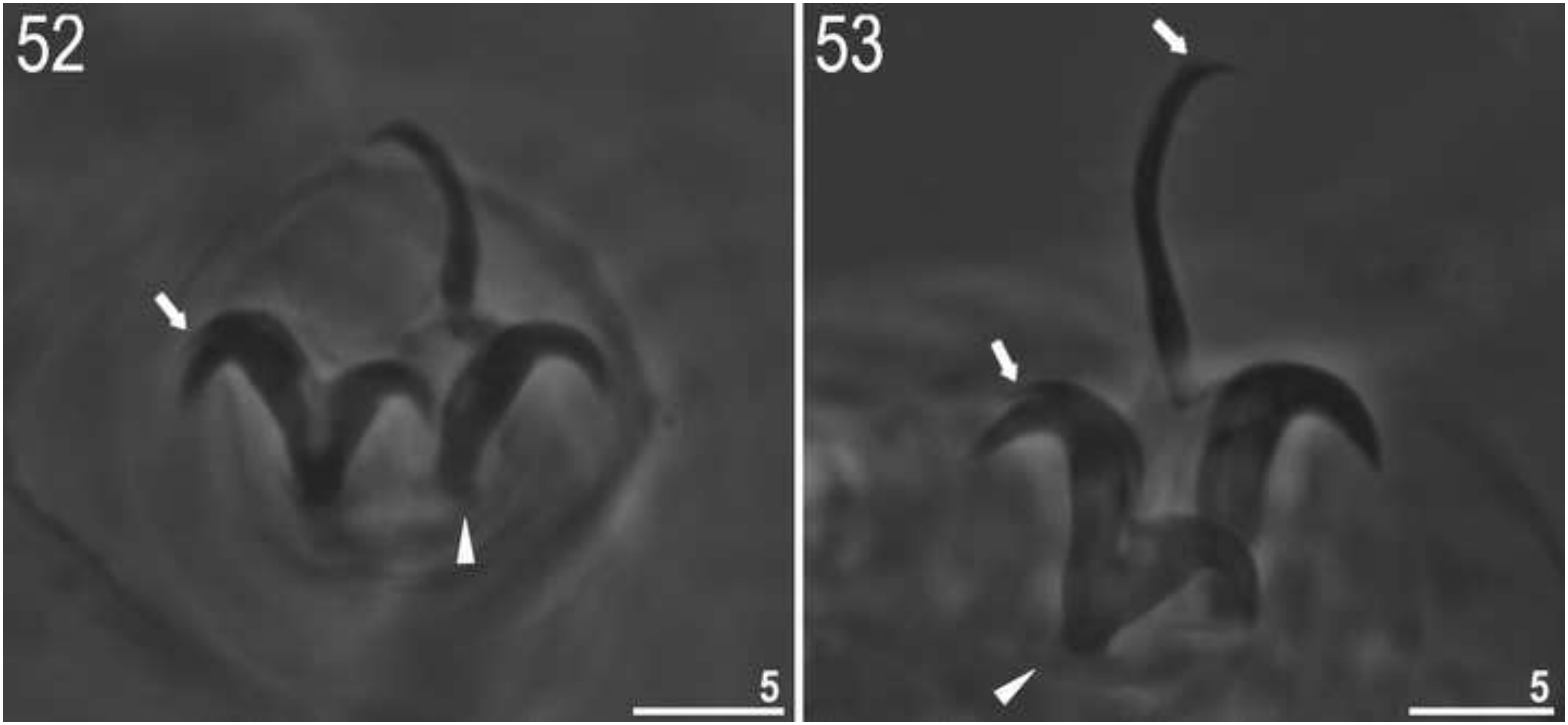

S1 Table

Click here to access/download
Supporting Information
S1_Table.xlsx

S2 Table

Click here to access/download
Supporting Information
S2_Table.xlsx

S3 Table

Click here to access/download
Supporting Information
S3_Table.xlsx

S4 Table

Click here to access/download
Supporting Information
S4_Table.xlsx

S5 Table

Click here to access/download
Supporting Information
S5_Table.xlsx

S6 Table

Click here to access/download
Supporting Information
S6_Table.xlsx
